# The transcription factor combination MEF2 and KLF7 promotes axonal sprouting in the injured spinal cord with functional improvement and regeneration-associated gene expression

**DOI:** 10.1186/s13024-025-00805-4

**Published:** 2025-02-08

**Authors:** Callan L. Attwell, Inés Maldonado-Lasunción, Ruben Eggers, Bastiaan A. Bijleveld, Ward M. Ellenbroek, Natascha Siersema, Lotte Razenberg, Dédé Lamme, Nitish D. Fagoe, Ronald E. van Kesteren, August B. Smit, Joost Verhaagen, Matthew R. J. Mason

**Affiliations:** 1 Laboratory for Regeneration of Sensorimotor Systems, Netherlands Institute for Neuroscience, An Institute of the Royal Academy of Arts and Sciences, Amsterdam, the Netherlands; 2 Department of Molecular and Cellular Neurobiology, Center for Neurogenomics and Cognition Research, Neuroscience Campus Amsterdam, Vrije Universiteit Amsterdam, Amsterdam, the Netherlands; 3 Division of Neuroscience, Department of Brain Sciences, Imperial College London, London, W12 0NN UK

**Keywords:** Axon regeneration, Transcription factor over-expression, AAV, Spinal cord injury, Promoter analysis, Neuron-intrinsic, Gene expression profiling, Transcription factor combinations

## Abstract

**Background:**

Axon regeneration after injury to the central nervous system (CNS) is limited by an inhibitory environment but also because injured neurons fail to initiate expression of regeneration associated genes (RAGs). The potential of strong RAG expression to promote regeneration in the CNS is exemplified by the conditioning lesion model, whereby peripheral nerve injury promotes regeneration of centrally projecting branches of the injured neurons. RAG expression could potentially be induced by delivery of the right set of transcription factors (TFs). We here aim to identify TF combinations that activate this program.

**Methods:**

We first analysed binding site motifs in promoters of the RAG program to identify nine candidate growth-promoting TFs. These were systematically screened in vitro to identify combinations that had potent neurite-growth promoting activity. Next, adeno-associated viral vectors were used to express these TF combinations in vivo in L4/L5 dorsal root ganglia to test whether they would promote regeneration in a spinal cord injury model (dorsal column lesion) in female rats. To determine whether they could activate the RAG program we carried out gene expression profiling on laser-dissected dorsal root ganglion neurons specifically expressing these TF combinations, and of DRG neurons that had been axotomized.

**Results:**

Promoter analysis identified ATF3, Jun, CEBPD, KLF7, MEF2, SMAD1, SOX11, STAT3 and SRF as candidate RAG-activating TFs. In vitro screening identified two TF combinations, KLF7/MEF2 and ATF3/KLF7/MEF2, that had potent neurite-growth promoting activity, the latter being the more powerful. In vivo, KLF7/MEF2, but not ATF3/KLF7/MEF2 or KLF7 or MEF2 alone, promoted axonal sprouting into the dorsal column lesion site and led to improved functional recovery. Gene expression profiling revealed that unexpectedly, the MEF2-VP16 construct used had little transcriptional activity in vivo, suggesting additional steps may be required to achieve full MEF2 activity. All combinations except MEF2 alone induced RAG expression mirroring that induced by axotomy to significant extents, while ATF3/KLF7/MEF2, KLF7 and ATF3, but not KLF7/MEF2 also induced apoptosis-related genes which may hinder regeneration.

**Conclusions:**

The TF combination KLF7/MEF2 partially mimics the conditioning lesion effect, inducing axonal sprouting into a dorsal column lesion and driving significant RAG expression, and also promotes functional improvement.

**Supplementary Information:**

The online version contains supplementary material available at 10.1186/s13024-025-00805-4.

## Introduction

Repair of the central nervous system (CNS) after injury fails, and one reason for this is that the injured CNS neurons fail to mount a robust regeneration-associated gene (RAG) expression program, in contrast to neurons of the peripheral nervous system [1–6]. Artificial induction of the RAG program is one approach to boost regeneration after CNS injury, and this might be achieved by delivering the appropriate transcription factors (TFs). However, identifying TFs capable of driving significant RAG expression in vivo, and thus regeneration, remains a significant challenge, partly because of the large number of regeneration-associated TFs that have been identified. For example, in our recently reported facial motor neuron dataset 60 TFs are upregulated in the first 4 days [7].

Delivery or activation of some of these TFs has had small positive effects on regeneration in dorsal root ganglion (DRG) neurons in the peripheral nerve, the dorsal root, or the ascending dorsal column. These include ATF3, SOX11, CREB, p53, STAT3, and SMAD1 [8–14]. However, these effects are much smaller than that achieved by a conditioning lesion, where full RAG program activation is achieved. In corticospinal neurons and retinal ganglion cells, increased axonal regeneration or sprouting has been induced with the transcription factors KLF6, KLF7 and SOX11 [12, 15–17], but JUN and STAT3 failed to promote corticospinal tract growth [18]. In all these experiments, it is not clear whether substantial RAG induction was attained, although some individual RAGs are induced by e.g. ATF3 [19]. Target genes of KLF6/7 have been identified but their relationship to the peripheral RAG program was not explored [17, 20].

TFs act co-operatively to regulate target promoters and in many cases physically interact (reviewed in [21, 22]). A potentially powerful approach therefore is to express combinations of key transcription factors that regulate the RAGs, using viral vector delivery. We have previously attempted this in vivo in DRG neurons [23], using a combination of ATF3, JUN, STAT3 and SMAD1. While this increased the speed of regeneration in the dorsal root, no effect was seen after spinal injury and the combination did not outperform ATF3 alone. So far this approach has not been applied to CNS neurons in vivo, although in vitro, a similar lack of synergy was seen when Jun and STAT6 were co-expressed in a cortical slice culture assay [24]. In vitro, co-expression of Jun and ATF3 did result in synergistic growth increases in DRG neurons and in cortical neurons when a tethered dimer was used [25].

The use of combinations of transcription factors has been applied with great success in the field of cell-type reprogramming [26, 27]. The transition from non-regenerating to regenerating neuron involves of the order of a thousand genes, probably fewer than change in many reprogramming transitions. The success of cell-type re-programming with relatively small numbers of TFs indicates that such an approach might work in peripheral neurons to induce the regenerative state. In DRG neurons it is clear that the regeneration program is accessible with the right stimulus. However, given the large number of TFs with an implicated role in the transition, and allowing that a combination of multiple TFs might be necessary, the search space for the right combination becomes potentially very large.

Several attempts have been made to identify key TFs that can promote regeneration by gene network analysis, promoter analysis of the RAG program and epigenetic profiling [17, 18, 28–36]. These approaches frequently identify the well-known factors AP1, STAT3, and SMAD family, and have generated a number of new candidate TFs but have so far not yielded additional factors with a demonstrable ability to promote long-distance regeneration.

Here we have taken a focused bioinformatics approach using a promoter analysis algorithm we recently developed on a gene expression dataset from regenerating motor neurons from Jun-knockout mice [7]. We have analysed the promoters of the RAGs from this same dataset to identify nine key TFs that potentially regulate the RAG program, including the novel factor MEF2. We then carried out combinatorial screening in a DRG-neuron like cell line to identify combinations of TFs that synergistically promote neurite outgrowth. We then applied these combinations to a spinal cord injury model in female rats in vivo and show that one of them, KLF7/MEF2, promotes functional recovery and axonal sprouting into and around the lesion site, in contrast to either factor acting alone. Curiously, the addition of ATF3 to this combination was beneficial in vitro but abolished its positive effects in vivo. Furthermore, we performed gene expression profiling on DRG neurons over-expressing these TFs and combinations in vivo, to precisely determine the effects of the TFs we delivered on gene expression, and relate this to axotomy-induced RAG changes. All combinations except MEF2 alone promoted large scale axotomy-like changes in gene expression. This analysis revealed a set of likely targets for ATF3 and KLF7, and indicated that these TFs have overlapping target genes. Furthermore, although the MEF2 construct had low transcriptional activity by itself, it changed the targets of KLF7 sufficiently to promote functional recovery and sprouting.

## Methods

### Identification of candidate transcription factors

The promoters of RAGs expressed in regenerating mouse facial motor neurons (FMN), 1 day after axotomy, were analysed to identify key TFs which might regulate these RAGs. RAGs were identified from phenotypically wild-type mouse FMNs [7] and their promoters were compared with the promoters of genes which were expressed above median level but not regulated (no significant regulation and fold-change below 1.3 fold).

Transcription factor motifs over-represented in RAG promoters were identified using the algorithm described previously in [7], which scores sequences using position weight matrices for TF binding site motifs and uses a flexible scoring threshold to optimize the over-representation ratio between a target promoter set and a control promoter set, and incorporates conservation across species in the scoring process.

TF binding site position weight matrices were from the TRANSFAC public database [37] and JASPAR 2020 CORE and PBM collections [38]. Conservation across species was such that binding sites had to match in at least 5 out of 15 species. Promoter lengths of 200–1000 bp were used, with the best-scoring length used for each motif. Significance of over-representation was determined with a binomial test. Thresholds of *p* < 0.001 and an over-representation ratio of 2 were applied. False discovery rate was calculated by resampling of the input promoter sets. Related motifs were then grouped together if they occurred at the same locations (for at least half the sites of either motif).

TFs indicated by over-represented motif groups were then filtered, first on whether they are expressed in both mouse FMN and rat DRG neurons; then whether either: they are upregulated after axotomy in both mouse FMN and rat DRG neurons, or a post-translational activation mechanism is known to exist, that is activated after axotomy (see Supp. Tables S1, S2). Expression and upregulation in mouse FMN was determined from the dataset of [7] and expression/upregulation in rat DRG neurons was determined using the dataset of [36]. Motifs for factors with closely related molecules, including Smad, Sox and Klf families, were taken to indicate all family members prior to filtering.

To identify predicted target genes of over-represented motifs, the analysis was re-run with threshold optimization for the ‘additional sites’ measure [7], since this identifies a greater number of potential target genes.

### Plasmids and viral vectors

We generated expression constructs containing the open reading frame (ORF) of each transcription factor selected for screening. Each ORF was inserted into the dual promoter adeno-associated viral vector (AAV) transfer vector pAGLWFI [39]. This vector expresses a gene of interest and co-expresses GFP that has an added farnesylation signal to promote axonal transport. A ‘No-TF’ vector was made that expressed GFP only, using pAGLWFI with the second expression slot left empty.

The sequences used are given in Table 1:

**Table 1:** Sequences of transcription factors used for overexpression

Factor	Form	Species	Source	Reference
ATF3	wild-type	rat	IMAGE clone 7,100,767	
CEBPD	wild-type	mouse	RIKEN clone I830043N22 / MGI:3,569,106	
JUN	wild-type	rat	IMAGE clone 7,124,370	
KLF7	wild-type	mouse	IMAGE clone 3,499,191	
MEF2	MEF2-VP16	mouse	Gift from Dr. Eric N. Olsen	[40]
SMAD1	hSmadl-EVE	human	Addgene 22,993	[41]
SOX11	wild-type	mouse	IMAGE clone 5,716,171	
SRF	SRF-VP16		Gift from Dr. David Ginty (Originally from Dr. Ravi Misra)	[42].
STAT3	Stat3-C	mouse	Addgene 13,373	[26]

These plasmids were used for transfection in the neurite outgrowth assay and for AAV production. The production and titration of AAV serotype 5 vector particles was performed as described [43].

For the gene expression profiling of DRG neurons expressing TFs the dual promoter plasmids expressing KLF7, MEF2 and ATF3 were modified by replacing GFP with different fluorescent proteins. These were paired as follows: MEF2 with GFP (non-farnesylated); KLF7 with mCherry; ATF3 with mito-YFP (yellow fluorescent protein with a mitochondrial targeting sequence; from pEYFP-Mito, Clontech). An additional control plasmid containing no TF and GFP was also generated. AAV5 vectors were generated with these plasmids as above.

### Neurite outgrowth assays

F11 cells [44, 45] were seeded directly into a 96 well cell culture plate (Greiner) at 60–70% confluence in DMEM (Invitrogen, Bleiswijk, the Netherlands) containing 10% FCS. The next day the medium was changed to DMEM (Invitrogen) containing 2% FCS and after 2 h cells were transfected with the AAV dual vector plasmids (with 0.16 µg total DNA per condition for 96 wells plates) using lipofectamine 2000 (Invitrogen) in Optimem medium (Invitrogen) according to the manufacturer’s protocol. The following day single plates were split into three 96 wells plates (each well containing 92ul Neurobasal medium (Invitrogen) with 1 x B-27 supplement (Invitrogen), 1% Glutamax (Invitrogen), 1% PS (Invitrogen) and 6.25 µM cytosine arabinoside (Sigma) to kill dividing cells. The 60 inner wells were used while the outer wells contained water to prevent the inner wells from drying out. Cell density after passaging was approximately 1.000 transfected cells (based on counts of GFP -positive cells) per well. Cells were fixed by adding one volume of 8% PFA in 0.1 M phosphate buffer after 24 h, 48–72 h. In the additive screen at least 6 replicate wells per condition were generated on a minimum of 2 different plates. In the subtractive screens at least 4 replicate wells per condition were used. Neurite outgrowth was quantified using a Cellomics ArrayScan HCS Reader (Thermo Scientific, Pittsburgh, PA) using the Neuronal Profiling 3.5 algorithm to trace GFP positive neurites from F11 cells at 5x magnification.

Cells were identified as round objects in low exposure time images of GFP, while neurites were identified in higher exposure time images of GFP. We analysed the parameter ‘Neurite Total Length’, which is the total neurite length per cell.

### Experimental animals and surgical procedures

All experimental procedures and postoperative care were carried out with approval from the animal experimentation ethical committee of the Royal Netherlands Academy of Sciences. Female Wistar rats (9–12 weeks old; Harlan, Horst, The Netherlands) were used. Animals were housed under standard conditions with food and water ad libitum, and a 12-hour: 12-hour light/dark cycle. All surgery and functional testing were carried out by experimenters blinded to experimental group identities.

For the experiments with dorsal column injury and TF overexpression, there were six treatment groups (No TF, KLF7, MEF2, KLF7/MEF2, ATF3/KLF7/MEF2 and sham- operated animals) each with 9 animals. We previously tested the horizontal ladder and inclined rolling ladder with cervical dorsal column lesions [46], and power analysis based on these data indicated that *n* = 8 would allow detection of effect size of 60% of that seen in [46] with 80% power for both tests. Power analysis was carried out as in [46] with the simulation of 50% more steps for the inclined ladder to account for its increased length. An initial group size of *n* = 9 was chosen to allow for potential loss of 1 animal per group. One animal each from the MEF2 and No TF groups were euthanised before the time-course was completed leaving *n* = 8 for these two groups. All except sham-operated animals received a dorsal column lesion four weeks after viral vector delivery to the L4 and L5 DRGs. AAV vectors were injected into the left L4 and L5 DRG as described [47] under isoflurane anaesthesia. 1 µl was injected into each DRG. Total viral titre was matched for all groups, divided equally among constituents of combination groups, at 8.1 × 10^12^ GC/ml. The sham group also receiving the GFP-only vector. The total amount of GFP-expressing vectors in all groups was therefore 8.1 × 10^9^ GC per DRG.

The dorsal column lesions were performed as follows. Animals were anesthetized using isoflurane. Following an incision along the dorsal midline, a laminectomy at C4 was performed to expose the spinal cord and the dura mater was opened as in [46]. To minimize compression damage of the spinal cord we first inserted a 30G needle at 1 mm lateral to the midline on either side to a depth of 1.6 mm. The resulting hole was then enlarged by inserting a 27G needle to the same depth. Finally the tips of a pair of microscissors were inserted into the same holes to the same depth and then closed, resulting in transection of the dorsal column. A small piece of subcutaneous fascia was placed over the lesion and a small amount of fibrin glue placed on top. Sham-operated animals received only the laminectomy. The muscles overlying the spinal cord were loosely sutured together with a 5 − 0 suture and the wound closed. Animals were allowed to recover at 37 °C and received postoperative analgesia (Temgesic 0.03 ml/100 g body weight s.c.; Schering-Plough, Maarssen, the Netherlands), and survived for twelve weeks after injury until perfusion.

Three days before perfusion, all animals were anesthetized with isoflurane and the left sciatic nerve was exposed. Animals were injected with 3 µl cholera toxin subunit B (CTB; 10 mg/ml) (103B, List Laboratories Inc., Campbell, CA) in the sciatic nerve to transganglionically label ascending dorsal column axons in the spinal cord.

To visualize TF expression from AAV vectors at the time-point corresponding to injury, for each viral vector group *n* = 3 additional animals received injections of the viral vector mixtures described above in the left L4 and L5 DRG. Animals were then allowed to survive 2 weeks.

To determine the effect of GFP and a conditioning lesion (CL) on axonal regeneration and/or retraction after dorsal column lesion (DCL), we used three groups of 6 animals (GFP/CL/DL, CL/DCL, DCL only). In the GFP/CL/DCL group viral vectors were injected into the left L4 and L5 DRG with dual vectors expressing only GFP (8.1× 10^12^ GC/ml) (as described above), 4 weeks before the DCL. The GFP/CL/DCL and CL/DCL groups received a CL 7 days prior to DCL. For the CL, animals were anaesthetized with isoflurane, the left sciatic nerve exposed and transected at the mid-thigh level, followed by wound closure. All groups received a DCL and transganglionic tracing with CTB as described above. Where a CL was given, CTB was injected into the proximal stump. Anaesthesia and pain relief were administered as described above.

At all experimental endpoints for the above experiments, animals were injected with a lethal dose of pentobarbital and transcardially perfused with 0.9% saline followed by 4% paraformaldehyde (PFA) in phosphate buffer. Brain stems, spinal cords and DRG were post-fixed in 4% PFA for 3–4 h at room temperature, transferred to 30% sucrose in phosphate-buffered saline, and were frozen in Tissue-Tek OCT (4583; Sakura Finetek Holland) the following day.

Six groups of rats (*n* = 4) were used for gene expression profiling of TF-expressing neurons. AAV vectors delivered to each group were: No TF/GFP (control); MEF2/GFP; KLF7/mCherry; ATF3/mitoYFP; KLF7/mCherry and MEF2/GFP; KLF7/mCherry, MEF2/GFP and ATF3/mitoYFP. The given combinations of titre-matched viral particles were injected directly into the left L4 and L5 DRGs as above. The total titre injected was in all cases 8 × 10^9^ GC per DRG in a volume of 1 µl. For TF combinations, total titre was divided equally among the different viral vectors. Animals were allowed to survive for four weeks after injection of AAV.

Three groups of rats (*n* = 4) were used for gene-expression profiling of axotomized DRG neurons: animals that received no injury; and two groups of animals that received sciatic nerve injury, with 1 day and 7 day survival. *N* = 4 was chosen as this gives sufficient power to detect most differentially expressed genes with the chosen read-depth of 30 million [48]. Animals were anaesthetized with isoflurane, the left sciatic nerve exposed and transected at mid-thigh level. CTB-Alexa Fluor 594 and Fluoro-ruby were injected at the lesion site into the proximal stump, to retrogradely label injured neurons cell bodies for subsequent laser dissection.

The animal groups for all experiments with numbers used are also given in Supp. Table S3. All animals were euthanized with a carbon dioxide/oxygen mixture. The left L4/5 DRGs were dissected, embedded in O.C.T. Compound (Sakura SAK 4583) and frozen on dry ice. The blocks were stored at -80ºC until processing. Tissue processing, laser dissection, RNA extraction and gene expression profiling are described in section ‘RNA Sequencing’ below.

### Immunohistochemistry

Immunohistochemical staining with GFP, CTB and GFAP was performed to visualise ascending dorsal column axons of transduced L4/L5 DRG neurons following dorsal column lesion. Longitudinal sections of cervical spinal cords were cut at 20 μm thickness on a cryostat in two series and mounted with water on Superfrost Plus glass slides (Menzel-Glasser, Braunschweig, Germany). Sections were fixed with 4% PFA for 5 min and blocked with blocking medium (2% horse serum, 0.2% triton X100 in 1xTBS) for 1 h.

Primary antibodies were as follows. Goat anti-CTB (1:100,000 with 72 h incubation) (List BIological Laboratories); rabbit anti-GFP (1:1000, Abcam; ab290); mouse anti-GFAP (1:4000, Sigma G3893). Secondary antibodies were: biotinylated horse anti-goat (1:300, Vector Laboratories); donkey anti rabbit Alexa488 and donkey anti-mouse Alexa647 (both 1:600, Jackson Immunoresearch). CTB signal was visualized and enhanced by applying the ABC kit (1:200, Vector) followed by biotinylated tyramide (1:400, PerkinElmer) and strepatavidin Cy3 (1:400, Jackson Immunoresearch).

Thoracic spinal cord sections were sectioned and stained for GFP and CTB. CTB staining was carried out as above, then sections were stained with chicken anti-GFP (1:500, Millipore) followed by donkey anti-chicken Alexa488 (1:600, Jackson Immunoresearch).

TF overexpression was visualized in DRGs using the following antibodies: rabbit anti-KLF7 (1:100, HPA030490, Sigma); rabbit anti-VP16 for MEF2-VP16 (1:3200, Ab4808, Abcam); rabbit anti-ATF3 (1:400, sc188, Santa Cruz Biotechnology). These were followed by biotinylated anti-rabbit secondary. KLF7 and MEF2 staining was enhanced using tyramide signal amplification as described above. Signal was then visualized with ABC kit (1:200, Vector) and 3,3’-Diaminobenzidine (DAB). Prior to sectioning and staining, whole DRG were treated by incubating in Tris buffer pH 9 at 65 °C for 3 h to effect antigen retrieval.

TF overexpression in HEK cells was visualized using the primary antibodies for KLF7, VP16 and ATF3 listed above at concentrations of 1:100, 1:100 and 1:400 respectively, and the following antibodies: anti-SMAD1 (1:800; sc7965; Santa Cruz Biotechnology), anti-c-Jun (1:200; sc1694, Santa Cruz Biotechnology), anti-SRF (1:800 Santa Cruz, SC-335), anti-SOX11 (1:300; sc20096 Santa Cruz Biotechnology) or anti-STAT3 (1:400; sc482; Santa Cruz Biotechnology). Primary antibodies were followed by Alexa594 labelled secondary antibodies. Quantification of co-expression of TFs and GFP in HEK 293T cells was carried out by automated image segmentation and quantification in ImageJ, with a manually determined threshold for GFP and a positive threshold for TF staining chosen such that < 2.5% of cells in control cells were positive.

### Histological quantification

Following dorsal column injury with TF overexpression, for quantification of axon growth at the lesion site, sections were photographed at 10x magnification with an Axioplan microscope (Zeiss). Quantification was performed by placing a grid over the images using ImagePro Plus (Media Cybernetics, Bethesda, MD, USA) with fixed intervals from − 3 mm to + 2 mm (positive distance indicating growth past the lesion site). The vertical counting line of the zero point was placed at the caudal-most boundary of the lesion, determined by identifying GFAP immunoreactivity. A blinded observer counted the GFP and GFP/CTB labelled fibres that crossed the grid lines. Section images were then manually aligned, using only the GFAP staining, in Adobe Photoshop to generate maximum intensity projections.

For quantification of retraction, the spinal cord caudal to the sections taken containing the lesion was also immunostained and imaged and axons were counted as described above in fixed intervals of 1 mm up to -20 mm. Retraction distances were calculated using the distance between the ‘leading edge’ to the caudal end of the lesion. The ‘leading edge’ was identified as the midpoint of the main body of retraction bulbs. The average distance of 3 consecutive sections with the most axons was taken for each animal.

Quantification of axons in the intact dorsal column was carried out in the axon tract caudal to the retracting axons (‘leading edge’). A blinded observer counted the total number of GFP and GFP/CTB labelled fibres at a fixed distance caudal to the leading edge. Quantification of axons in animals that received conditioning lesions was carried out as described above at distances from + 2 to -9 mm relative to the proximal lesion border.

Quantification of axonal sprouting in T8 cord was carried out on confocal images of GFP immunostained sections acquired over an area of 1.16 mm x 0.39 mm (3 frames). All sections containing GFP-positive axons were imaged. Images were pre-processed with a band-pass filter in ImageJ and then axon lengths were quantified using the software package Neuromath [49], with local noise threshold activated. For normalization, GFP-positive axons were counted in transverse sections of T10 spinal cord in sections double labelled with GFP and TuJ1 (1:500; Covance). Axon lengths obtained from Neuromath were normalized to the mean of T10 GFP-positive axon counts and counts obtained on longitudinal sections caudal to the lesion (see above) to give a number representing µm of growth per GFP-positive axon.

### Functional testing

All functional tests (described below) were performed one and two weeks before injury to get a baseline measurement, followed by weekly measurements after injury for eleven weeks. Three experimenters carried out the tests (CLA, WE & BAB), working in pairs, and each test was carried out consistently by the same experimenters throughout the time course (CLA present in all tests). Testers were blinded with regard to which animals were lesioned or sham-lesioned and which TF treatment injected animals received. The different tests were all performed in the same order each week and at the same approximate time of day. Animals had a daily pre-training period of 2 weeks prior to baseline measurements and all had mastered accurately traversing the respective platform/ladder and were comfortable being handled. In the horizontal ladder and inclined rolling ladder, any indication of mis-stepping including ‘stutter steps’ was counted as a slip.

The horizontal ladder, adapted from the gridwalk test [50] is a 0.9 m long horizontal ladder with a diameter of 15.5 cm. The rungs of the ladder are adjustable with a possible gap of 3.5–5.0 cm and were randomly adjusted for each time point to prevent a learning effect. Three runs per animal were video recorded and analysed by an independent blinded observer. Slips or misses and successful steps were recorded for the left and right hindlimbs and forelimbs. Counts of slips or misses and successful steps were used directly for statistical analysis, while for plotting the total number of slips and misses was divided over the total number of steps for each run and averaged for three runs to calculate the mean error ratio.

The inclined rolling ladder used in this experiment is a modified version of the ladder described in [46]. This contains rungs which are half smooth (rolling) and half rough (and fixed) rungs, intended to simultaneously test texture sensation and proprioception, which depend on the ascending dorsal column. The ladder was modified with a transparent entry box and runway which stimulated the animals to ascend to the darkened home box at the top, and was made longer with 12 rungs instead of 8, for more data points per run. To prevent a learning effect, the orientations of the ladder rungs were randomized at each time point. Three runs per animal were video recorded and analysed by an independent blinded observer. Successful steps, slips and the type of rung from which these occurred (rough or smooth) were scored. The slips measure was used, defined as slips vs. successful steps on smooth bars only. Counts of successful/failed steps per run by these definitions were used directly for statistical analysis. For plotting, the mean error ratio for the slips measure was calculated as the number of slips divided by the total number of steps on smooth rungs for each run and averaged for three runs.

The CatWalk XT gait analysis system [51, 52] was used. Three runs per animal were recorded. All four paws were automatically labelled using the Catwalk software and were checked afterwards by a blinded experimenter for gait analysis. For each animal the base of support, stride length, swing time, print width, mean pixel intensity and maximum contact area for the hind paws were measured using the CatWalk software package. These parameters were chosen based on previous literature describing dysfunction after SC lesion [46, 53] and because they could be expected to partially depend on proprioceptive function. Mean values of left and right hind paws were taken. All values were normalized to baseline measurements for each animal for plotting.

### RNA sequencing

DRG that had been injected with AAV vectors expressing TF and fluorescent protein were cryosectioned at 20 μm thickness onto polyethylene-naphtalate (PEN) membrane slides (Carl Zeiss, Germany) and left to dry at room temperature for 1 h before storage at -80 °C. DRG sections were dehydrated prior to laser dissection for 5 s in 70% EtOH, 2.5 min 90% EtOH, rinsed in 100% EtOH, 3 min in 100% EtOH on ice and finally air dried on foil immediately. Laser dissection was carried out using a PALM MicroBeam (Carl Zeiss, Germany) and PALM Robosoftware. Fluorescent filters were used for identification of fluorophore- or tracer-labelled cells. Large diameter (> 40 μm) neurons were selected using the measurement tool and cutting margin of 10–20 μm was left around each neuron. Following laser cutting, the laser was used to catapult neurons into an adhesive cap. An average of 184 neurons were collected per animal (range 80–255). Trizol Reagent was added to the samples prior to storage at -80ºC.

RNA isolation was performed with the RNeasy^®^ Micro Kit (QIAGEN, Germany) following an adapted version of the manufacturer’s protocol. Chloroform/EtOH RNA extraction of RNA from Trizol Reagent cell lysate was performed in Phase-Lock Gel (PLG) tubes (heavy 1.5 ml; 5 PRIME, Gmbh), followed by the RNeasy protocol. RNA was eluted with 15 µl of RNase-free MilliQ water. RNA samples were then cleaned up by extraction with water-saturated 1-butanol followed by water-saturated ether [54]. Samples were then processed by GenomeScan, Leiden, the Netherlands for determination of yields, further assessment of quality and RNA sequencing. Single-end reads were generated with a read-depth of 30 million. All samples had a RNA Quality Number > 5.2 (determined using a Fragment Analyzer, Agilent Technologies, Santa Clara, CA). Average RNA input per sample was 14.5ng.

The NEBNext Ultra Directional RNA Library Prep Kit for Illumina was used to process the sample. The sample preparation was performed according to the protocol “NEBNext Ultra Directional RNA Library Prep Kit for Illumina” (NEB #E7420S/L). rRNA was depleted from total RNA using the rRNA depletion kit (NEB #E6310) and then mRNA was isolated using oligo-dT magnetic beads. After fragmentation of the RNA, cDNA synthesis was performed. This was used for ligation with the sequencing adapters and PCR amplification of the resulting product.

The quality and yield after sample preparation was measured with the Fragment Analyzer (Agilent Technologies). The size of the resulting products was consistent with the expected size distribution (a broad peak between 300 and 500 bp). Clustering and DNA sequencing using the Illumina Nextseq 500 was performed according to manufacturer’s protocols. A concentration of 1.6 pM of DNA was used. Nextseq control software v2.0.2 was used. Image analysis, base calling, and quality check was performed with the Illumina data analysis pipeline RTA v2.4.11 and Bcl2fastq v2.17.

### RNA sequencing analysis

The rat transcriptome, based on rat genome version Rnor_6.0 was obtained from Ensembl (release 87). cDNA and non-coding RNA transcript data were combined and AAV vector elements were added to create a combined reference transcriptome. The cDNA transcriptome was first filtered to remove transcripts annotated as pseudogenes, nonsense-mediated decay, non-stop decay or retained-intron. Reads were aligned to the rat transcriptome using TopHat2 [55]. Reads were then counted and converted to gene-level counts using featureCounts [56] with multi-mapping reads and fractional read counting enabled.

All subsequent analysis was carried out in the statistical programming environment R. Principle component analysis was performed on the 500 most variable genes after variance stabilisation using DESeq [57]. Counts were analysed for differentially expressed genes using DESeq. Only genes where at least 3 samples had non-zero counts over the whole experiment were used (leaving 15995 genes). After this filtering step, the sciatic nerve injury groups and the AAV-injected groups were analysed separately, although an analysis of all groups was also carried out for the No-TF vs. naïve uninjured comparison. For the sciatic nerve injury group, all samples were compared with the uninjured DRG neurons. For the AAV–injected groups, all samples were compared with the AAV-No-TF injected samples. A significance threshold (false discovery rate) of 0.05 was used. Fold-changes for plotting and subsequent analysis were calculated using log_2_ of counts per million, with 1 added to the counts to avoid taking logs of zero.

For the cluster heatmap and fold-change correlation analysis, log fold-changes for the mean of each group were calculated with respect to the relevant control. In other words, the nerve injury groups log fold-changes were calculated with respect to the uninjured DRG, whereas for the AAV-TF treated DRG log fold-changes were calculated with respect to the AAV-GFP only group. The cluster heatmap was generated using log fold-changes by standard hierarchical clustering by Euclidean distance for genes and group means, using base R. Rank-rank hypergeometric overlap analysis [58] was performed using R package *RRHO* [59], using signed log *p*-values from the differential expression analyses for ranking. *P*-values were corrected for multiple testing using the Benjamini-Yekutieli method before log transformation and plotting.

Weighted Gene Correlation Network Analysis (WGCNA) was carried out on log fold-change values as described above. All genes where at least 3 samples had non-zero counts were used. Default parameters were used apart from the ‘soft power’ parameter which was determined by the recommended method [60]. After clustering, only genes with cluster membership (correlation to eigengene) over 0.7 were kept. Clusters were plotted using the mean log fold changes genes in the cluster calculated for each sample.

Gene ontology (GO) annotations were obtained from geneontology.org (all rat gene annotations) and EMBL-EBI QuickGo (for ncRNAs). GO analysis was carried out with R package *TopGO* [61], using the ‘parent-child’ algorithm with Fisher’s exact test. A *p*-value cut-off of 0.01 was used, and GO classes were required to have a minimum over-representation ratio of 2, were required to be at least 4 nodes away from the base GO class (‘all’), and to contain at least 2 genes and at least (log(n)- 0.5) genes for an input of n genes. False discovery rate was calculated by resampling. Over-represented GO classes with a parent-child relationship were grouped together to create groups of related over-represented classes.

Gene-set enrichment analysis (GSEA) was carried out using the GO BP ontology as the target gene sets using R package *fgsea* [62] and *clusterProfiler* [63] for plotting.

### Statistical analyses

All statistical analysis was carried out in the statistical computing environment R [64]. All data are plotted as mean ± standard error of the mean unless otherwise stated.

For the F11 neurite outgrowth assay, average total neurite outgrowth per well was used. To account for baseline growth differences between plates, conditions were compared using a linear mixed model with the inclusion of plate identity as a random effect, with R package *nlme* [65]. The data were found to be normally distributed by performing a quantile-quantile plot of the residuals of a linear mixed model of all conditions. Dunnett’s post-hoc test was used to compare each condition to the relevant control. Neurite outgrowth was plotted using the effect of each condition as determined by the linear mixed model (i.e. with the plate effect corrected for) using R package *effects* [66]. Synergistic effects were calculated using predicted effect values from the linear models, as follows. First values for TF-induced outgrowth were calculated for each condition by subtracting baseline growth (outgrowth in the GFP condition). Synergistic growth increase was calculated as the increase in induced neurite outgrowth from adding an individual TF to a given set of TFs, over the sum of outgrowth values obtained from the individual TF (on its own) and the recipient set.

Axonal sprouting at the lesion was analysed as follows. Normalized total axon length at the lesion site was calculated as the area under the curve of axon counts vs. distance, from − 2.0 mm to + 2.0 mm, divided by the axon count at -3 mm, and was compared between groups using a linear model F-test with Tukey’s post-hoc test. Where no axons were present at -3 mm or closer, axon length at the lesion site was counted as zero. Retraction distances were compared using a linear model F-test with Dunnett’s post-hoc test. Sprouting in thoracic cord was compared using a linear model F-test using the White-Huber adjustment for heteroscedasticity using R package *car* [66]. Post-hoc comparisons for this model were carried out using R package *multcomp* [67], with heteroscedasticity-corrected covariance matrices. Intact caudal dorsal column axon counts of GFP and co-labelled GFP/CTB fibres were compared across groups using a linear model F-test.

For the horizontal ladder and inclined rolling ladder, data were analysed using a binomial generalised linear mixed model (GLMM), with operated status and group/operated interaction as fixed effects, animal as random effect (intercept and slope) and time as a covariate, using R package *lme4* [68]. Where overdispersion was indicated an observation-level random effect was added. Right-paw error ratio, transformed with the logit function, was included as a covariate for the horizontal ladder. *p*-values were calculated by the parametric bootstrap method in R package *pbkrtest* [69], as in [46]. Post-hoc comparisons were carried out by using GLMMs with 2 groups (TF treatment vs. GFP control), with *p*-values being adjusted for multiple comparisons using the Benjamini-Hochberg method. Average effects of each group over time courses were determined using R package *effects* [66, 70]. For the catwalk, data were analysed using linear mixed models using package *nlme*.

For correlation analysis of fold-changes in the RNASeq data, mean fold-changes for each gene were calculated for each group and the fold-changes were correlated using Pearson’s correlation coefficient. Correlations of expression fold-changes of transcription factor predicted target genes induced were assessed using individual level data, using linear mixed models with random effects for animal and gene using R package *lme4* [68] and *lmerTest* [71].

## Results

### Promoter analysis of a RAG dataset reveals 9 key TFs

**Fig. 1:**
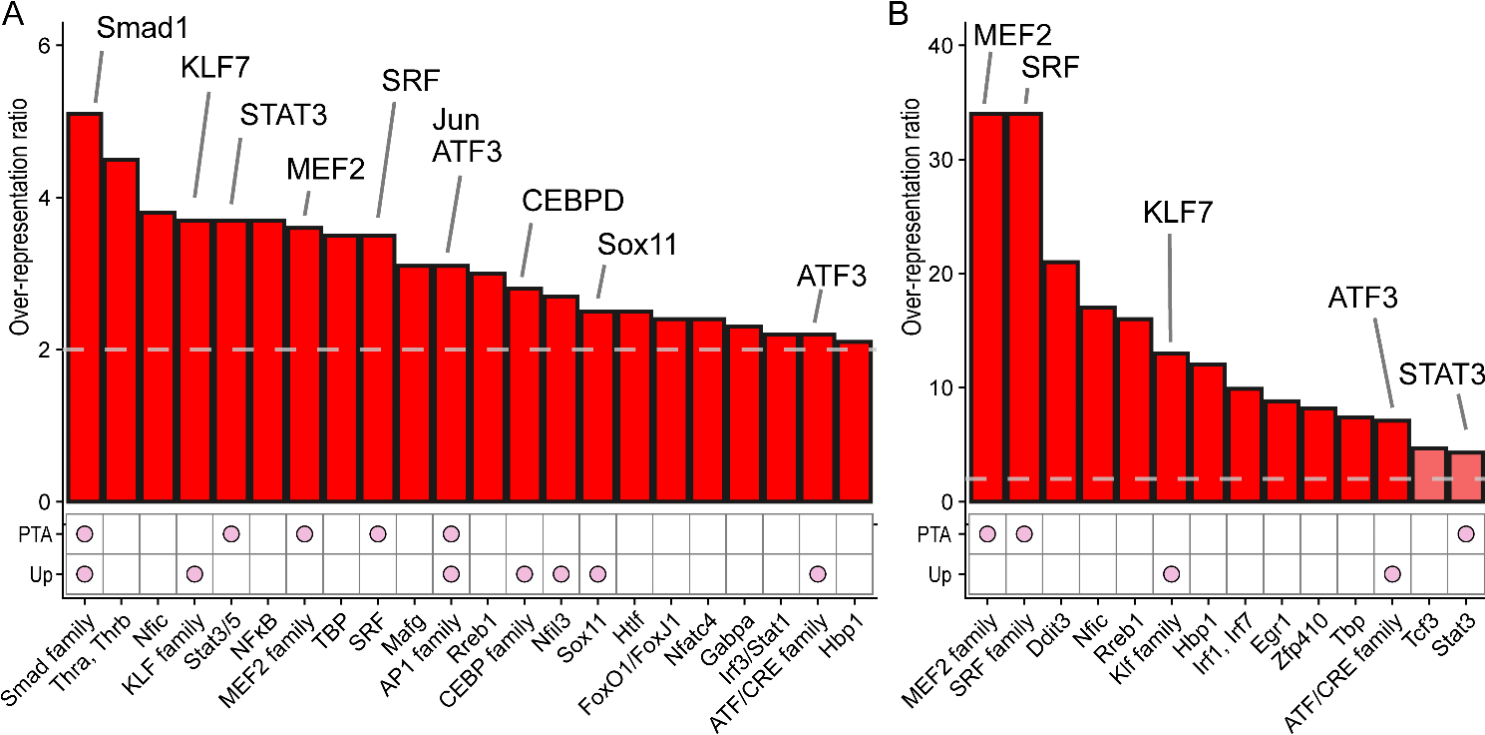
Analysis of regeneration associated gene (RAG) promoters identifies 9 key transcription factors. The promoters of a set of RAGs were analysed to identify key transcriptional regulators. The RAGs analysed were the set of genes upregulated in regenerating mouse facial motor neurons (FMN) 1 day after axotomy [7]. Promoter sequences were compared with the promoter sequences of genes that were not regulated, and scores were generated for binding site motifs using position weight matrices from TRANSFAC and JASPAR 2018. Scores were calculated to incorporate cross-species conservation and thresholds were chosen to maximize the ratios of motif occurrence frequencies between the two groups. The upper graphs show factors with over-represented binding site motifs and their maximized ratios. The grid below shows additional criteria for selection: ‘Up’ indicates whether the TF is upregulated after axotomy both in mouse FMN and in rat dorsal root ganglia, PTA indicates the existence of a known Post-Translational Activation mechanism activated by axotomy. **(A)** The analysis for all day 1 FMN RAGs compared with unregulated genes. This resulted in a selection of 9 TFs for subsequent screening, shown above the graph. ATF3 appears twice because it binds two different TFBS families (CRE and AP1). **(B)** Analysis for the promoters of only the TFs among the day 1 FMN RAGs, compared with unregulated genes. Notably, over-representation ratios are much higher when considering only TF promoters, and MEF and SRF show particularly strong signals. After applying the same additional criteria for upregulation or post-translational activation, this identifies a subset of the factors found in Fig. 1A, but with substantially higher over-representation ratios, confirming the potential importance of these 5 factors. False Discovery Rate was < 0.1 for all red bars

We first carried out an analysis of RAG promoters to identify key TFs involved in regulating the RAG program. We used an algorithm we developed which uses cross-species conservation and a flexible scoring threshold to optimize TFBS motif over-representation [7]. RAG promoters from regenerating mouse facial motor neurons were compared with promoters of genes that were unregulated in these neurons after axotomy. We then cross-referenced the results with expression data for TFs from the mouse FMNs and another dataset of gene expression in rat DRG neurons after axotomy [36]. TFs that potentially bind the over-represented motifs were then filtered, being kept only if they were expressed in both mouse FMNs and rat DRG (Fig. 1A). A further filtering step was applied for evidence of activation after axotomy, such that a TF was kept only if it was upregulated in both models, or a known post-translational activation mechanism exists that is activated by axotomy (see Methods for further details). The results are summarized in Fig. 1A and shown in Supp. Table S1. Nine TFs were selected by this process: ATF3, CEBPD, JUN, KLF7, MEF2, SMAD1, SOX11, STAT3, and SRF. This list was the result of the application of the criteria listed above with the following exceptions: although KLF6 was indicated, KLF6 and KLF7 are highly similar and we chose KLF7 because of its previously shown effects on regeneration [12, 15]. NFIL3 was indicated but excluded because it was previously shown to be have a negative effect on regeneration [72], and CREM was indicated but not included since it binds the CRE site and we have a strong indication for ATF3 which already targets this site.

In a second analysis, we considered the promoters only of TF genes upregulated by axotomy, as we reasoned that TFs that regulate other TFs are more likely to be master regulators or hub TFs. This analysis is shown in Fig. 1B and Supp. Table S2, and after applying the same filtering criteria, we identified MEF2, SRF, KLF7, ATF3 and STAT3, a subset of the factors found in Fig. 1A. Notably much higher over-representation ratios are found with this approach, and MEF2 and SRF now show the strongest signals, suggesting one or both of these factors may be a hub in the RAG expression network.

For subsequent experiments, constitutively active forms of TFs were chosen where possible, in particular, STAT3C [26] and Smad1-EVE [41], while for SRF and MEF2 we used VP16 fusion proteins (SRF-VP16 and MEF2C-VP16) to maximize transcriptional activity. Dual promoter expression constructs were validated in HEK293T cells with immunocytochemistry (Supp. Fig. S1). Quantification of this immunocytochemistry showed that at least 90% of GFP-positive cells also expressed the co-delivered TF except Sox11 (78%) (Supp. Fig S1B). Since the vector backbone was the same for all TFs, this is likely due to the lower quality of antibody used for this TF.

### Combinatorial in vitro screening identifies ATF3, MEF2 and KLF7 as a synergising set of transcription factors

We next undertook a systematic search for synergistic combinations of these nine TFs with respect to neurite-growth promoting activity, by overexpressing them in F11 cells, a DRG-like cell line [44, 45]. Outgrowth at 72 h was used to compare conditions.

**Fig. 2:**
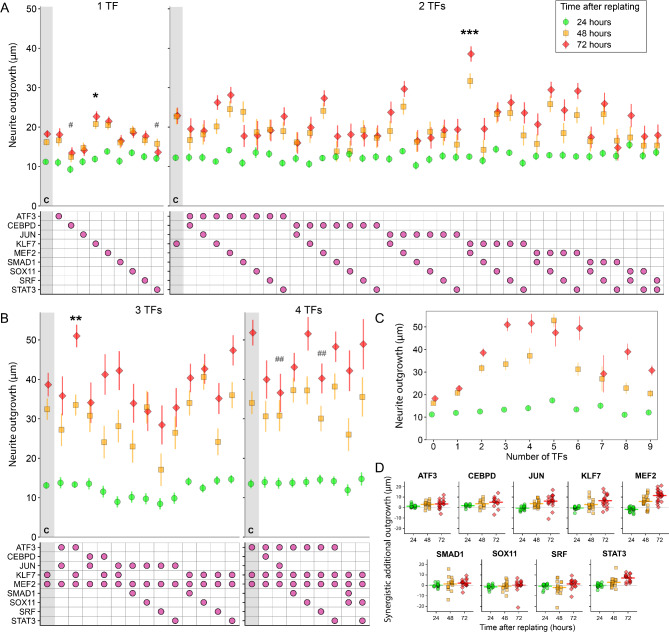
Combinatorial screen of 9 transcription factors for neurite growth promotion and synergy in F11 cells. F11 cells were transfected with dual promoter plasmids expressing GFP and a TF or the control vector expressing GFP only (No-TF), singly or in combinations. After 24, 48 and 72 h cells were fixed and automated microscopy and neurite tracing (Cellomics) was carried out on GFP-positive neurites and the total neurite length in µm calculated. Measurements at 72 h were compared using a linear mixed model with plate as a random effect and Dunnett’s post-hoc test. Shown are screens with 1, 2, 3 and 4 factors. Each time the number of factors was increased by one, combinations were compared with the best condition with one less factor. Control groups for each comparison are shown as the first condition, indicated by the letter C and grey background shading. The grid indicates which factors were present in each condition. **(A)** Screen of individual TFs and all pairs of TFs expressed together. Of the single TFs, KLF7 overexpression significantly increased total neurite length in comparison with GFP while CEBPD and STAT3 significantly reduced total neurite length. Among the co-expressed pairs, KLF7/MEF2 overexpression significantly increased total neurite length in comparison with KLF7. **(B)** Screen of selected combinations of 3 TFs and 4 TFs expressed together. ATF3/KLF7/MEF2 overexpression significantly increased total neurite length in comparison with KLF7/MEF2. No combinations of 4 factors are better than ATF3/KLF7/MEF2 while ATF3/JUN/KLF7/MEF2 and ATF3/KLF7/MEF2/SRF overexpression significantly decreased total neurite length in comparison with ATF3/KLF7/MEF2. **(C)** Maximum outgrowth achieved for each number of TFs in combination. Outgrowth begins to plateau with 3 TFs and starts to decline when more than 6 TFs are used. **(D)** Synergistic power of each TF. Each data point shows the synergistic additional neurite outgrowth (i.e. the growth increase beyond simple additive effects) at 72 h obtained by adding each individual TF to all other combinations that do not include it. All combinations from A-D and Supp. Fig. S2 were included. Horizontal bars indicated the mean. MEF2 and KLF7 show the greatest mean synergistic effects at 72 h. Key: *,# *p* < 0.05; **, ## *p* < 0.01; *** *p* < 0.001 (linear mixed model, Dunnett’s post-hoc test; *n* ≥ 6). Stars indicate increased outgrowth compared with the control condition, hashes indicate decreased outgrowth compared with controls

First, individual TFs were screened. KLF7 resulted in the largest increase in neurite outgrowth compared with the control (a 24% increase over GFP only), *p* < 0.05 (Fig. 2A), followed by MEF2-VP16, (n.s.; *p* = 0.07). Next, all possible pairs of TFs were screened and compared with the most potent single TF, KLF7. The pair with the greatest outgrowth was KLF7 and MEF2 (62% greater than KLF7 only; *p* < 0.001) (Fig. 2A). In the next round of screening, we took the two highest performing pairs, KLF7/MEF2 and JUN/MEF2, and combined each pair with the remaining seven candidate TFs to generate triple TF combinations. Neurite outgrowth for these combinations was compared with KLF7/MEF2 (the best performing pair). The resulting screen yielded the triple TF combination of ATF3/KLF7/MEF2 that induced significantly greater outgrowth than KLF7/MEF2 (a 29% increase; *p* < 0.01)(Fig. 2B). No other combination of three performed significantly better than KLF7/MEF2 (although KLF7/MEF2/STAT3 performed well). Finally, we combined this triple TF combination with the remaining six TFs, comparing the effects on neurite outgrowth with ATF3/KLF7/MEF2. None of the resulting combinations of four produced significantly greater neurite outgrowth than ATF3/KLF7/MEF2, and in fact two conditions, those containing c-Jun or SRF, performed significantly worse (*p* < 0.01; Fig. 2B).

We also carried out a subtractive approach, mirroring that of [26] (Supp. Fig. S2). Over-expression of all nine TFs did result in significantly higher neurite outgrowth than controls (*p* < 0.001), but this was not as much as that achieved by the most successful combination from the additive screen, ATF3, KLF7 and MEF2. TFs were subtracted from the set of 9 to identify those which had a negative effect on neurite outgrowth until 5 TFs remained. The results are shown in detail in Supp. Fig. S2. The maximum neurite outgrowth attained by any combination for each number of TFs is plotted in Fig. 2C. It can be seen that the neurite outgrowth promoting effect plateaus at 3 TFs. Increasing the number of TFs does not increase outgrowth further, while beyond 6 TFs the outgrowth starts to decrease. This combined additive and subtractive approach thus identified the triple ATF3, KLF7 and MEF2 as the optimum and most efficient combination to drive axon growth.

The synergistic power of each TF over the whole screening experiment was calculated and is shown in Fig. 2D. Each point represents the synergistic increase in neurite outgrowth obtained by adding the TF in question to a TF or a given combination of TFs, i.e. extra growth that is beyond a simple additive effect. At 72 h, MEF2 shows the greatest synergistic increases in outgrowth over all combinations, followed by STAT3 and KLF7. ATF3, JUN and CEBPD also show mostly positive synergistic effects. This indicates that in particular MEF2, and to a lesser extent KLF7, STAT3, ATF3, JUN, and CEBPD have positive synergistic interactions with most other TF combinations.

Example images of transfected F11 cells used for quantification from the strongest performing TF combinations KLF7/MEF2 and ATF3/KLF7/MEF2 and the control GFP are shown in Supp. Fig. S3.

### TF expression promotes sprouting and functional recovery after spinal cord injury

We next investigated the effect of the most potent TF combinations identified above on axon regeneration and functional recovery in vivo after spinal cord injury, using a dorsal column lesion model.

We overexpressed TFs using dual AAV vectors also expressing GFP [39]. These vectors were injected individually or in combinations into the left L4 and L5 DRG. The groups, designated by the TFs they received, were: MEF2-only; KLF7-only; KLF7/MEF2; ATF3/KLF7/MEF2; No-TF (i.e. AAV expressing GFP only). ATF3 alone was previously shown to have no effect in a dorsal column injury model and so was not included [19, 23]. Animals received a cervical transection of the ascending dorsal column. A sham-lesioned group received the No-TF vector.

**Fig. 3:**
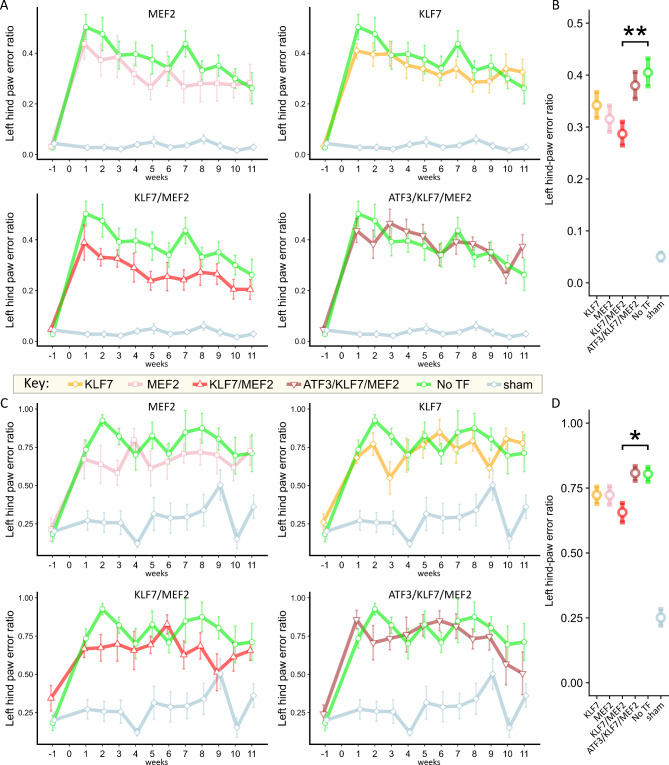
Combined KLF7 and MEF2 overexpression in dorsal root ganglion neurons leads to improved function after dorsal column injury. Animals were assessed for sensorimotor deficits over 11 weeks following a C4 dorsal column lesion, using a horizontal ladder (**A**, **B**) and an inclined rolling ladder (**C**, **D**). Animals were tested every week. Baseline measurements are the average scores at 1 and 2 weeks prior to the lesion. Mean error ratio is calculated as the total number of slips and misses of the left hind paw divided over the total number of steps for each run and averaged for three runs. **(A)** Time courses of error rates for each combination on the horizontal ladder in comparison with No-TF and sham groups. **(B)** Average effects of each group over the whole time course. KLF7/MEF2 perform significantly better than No-TF. **(C)** Time courses of error rates for each combination on the inclined rolling ladder in comparison with No-TF and sham groups. **(D)** Average effects of each group over the whole time course. KLF7/MEF2 perform significantly better than No-TF. * *p* < 0.05; ** *p* < 0.01 (binomial generalised linear mixed model; *n* = 8 for No TF, MEF2; *n* = 9 for other groups)

Animals were tested on the horizontal ladder, which is sensitive to deficits in hind-paw function after dorsal column lesion [46, 73]. In this experiment, deficits were detectable in No-TF treated animals up to 11 weeks following dorsal column lesion compared with sham-lesioned animals. Whole time-course comparisons show that KLF7/MEF2 treated animals made significantly less errors of the left hind paw (*p* < 0.01) compared with controls (No-TF). Overexpression of KLF7 only, MEF2 only or ATF3/KLF7/MEF2 did not result in any significant improvement compared with No-TF. The time-courses are depicted in Fig. 3A, and average effects over the time-course are given in Fig. 3B.

Forelimb function was also scored on the horizontal ladder (Supp. Fig. S4). Forelimb function was affected by the lesion to a lesser degree but remained stable across the time course in the TF-treated groups. In particular the KLF7/MEF2 group was very stable over the timecourse and similar to GFP, indicating the treatment effect was specific for the hindlimbs as expected. No significant differences were found between any TF group and the No TF group, although No TF vs. sham was significantly different (*p* < 0.001; binomial linear mixed model).

The second sensorimotor test was a modified version of the inclined rolling ladder described in [46]. Again, significant deficits were still detectable in No-TF treated animals up to 11 weeks following dorsal column lesion. In whole time-course comparisons KLF7/MEF2 treated animals made significantly less errors of the affected hind paw (*p* < 0.05). Overexpression of KLF7 only, MEF2 only or ATF3/KLF7/MEF2 did not result in any significant improvement compared with No-TF. The time-courses are depicted in Fig. 3C, and average effects over the time-course are given in Fig. 3D.

Animals were also tested with gait analysis using the Catwalk XT. For each animal the base of support, stride length, swing time, print width, mean pixel intensity and maximum contact area for the hind paws were analysed as these parameters were found to be sensitive to dorsal column lesion [46] or were reported in literature [74]. No significant changes were detected in any of the parameters, in any condition when compared with No-TF over the 11 weeks of measurements. However, while not significant, KLF7/MEF2 and to lesser degrees ATF3/KLF7/MEF2 and KLF7 only, appeared to show a trend towards an increased hind paw width in the base of support measurement (note this is a change away from sham group values).

**Fig. 4:**
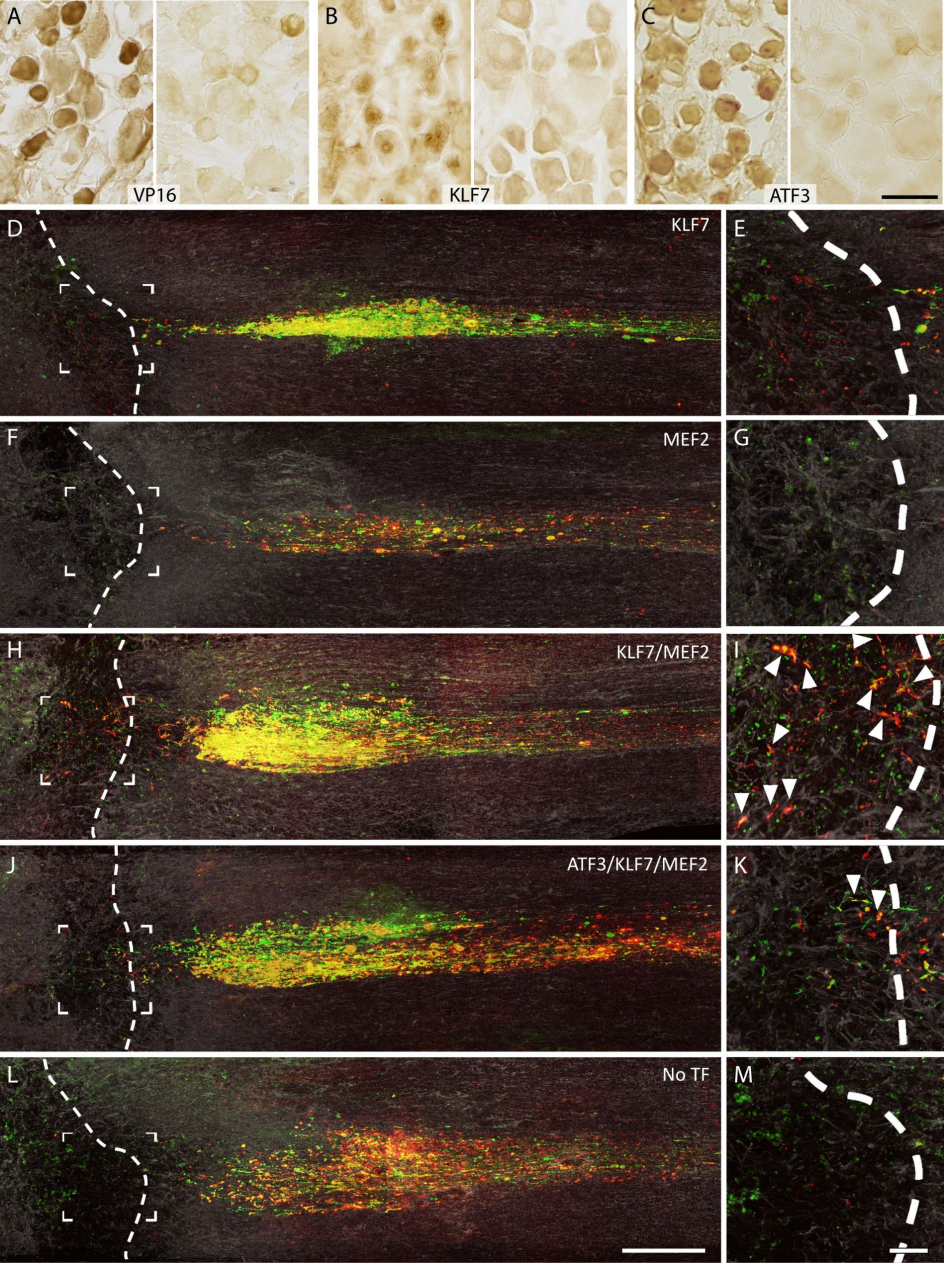
KLF7/MEF2 induces sprouting into and around the lesion after dorsal column lesion. **A**-**C**. Immunohistochemistry (IHC) for **(A)** VP16 (to visualize the MEF2-VP16 construct), **(B)** KLF7 and **(C)** ATF3 in dorsal root ganglia transduced with AAV vectors expressing these factors. Left panels are dorsal root ganglia injected with AAV expressing the TF in question, right panel are control DRG. **D**-**M** Ascending axons of dorsal root ganglion neurons after dorsal column injury, visualized by IHC for GFP (green), the transganglionic tracer CTB (red) and GFAP (grey) for one example animal from each group. Each image is a maximum intensity projection of images of all sections where CTB/GFP labelled fibres were found. The proximal lesion border is indicated by the white dashed line. **E**, **G**, **I**, **K**, **M** show magnifications of the boxed areas in **D**, **F**, **H**, **J**, **L** to show the lesion centre. Substantial differences in axon density are visible in the region proximal to the lesion, with a higher densities apparent in the animals of the KLF7 only, KLF7/MEF2 and ATF3/KLF7/MEF2 groups compared with the No-TF animal. In the animal of the KLF7/MEF2 group, axons can also be seen penetrating the lesion, nearly reaching the distal border (**I**, arrowheads). In the animal of the ATF3/KLF7/MEF2 group, a few axons enter the lesion but do not penetrate (**K**, arrowheads). No axons were seen entering the lesion in the other groups. Scale bars: **C**, 50 μm (applies to **A**, **B**, **C**); **L**: 500 μm (applies to **D**, **F**, **H**, **J**, **L**); **M**: 100 μm (applies to **E**, **G**, **I**, **K**, **M**)

Expression of the three TFs in DRG neurons was confirmed by immunohistochemistry (Fig. 4A-C). All three TFs were readily detectable by immunohistochemistry. KLF7 showed stronger nuclear staining, while MEF2 and ATF3 were both nuclear and cytoplasmic.

**Fig. 5:**
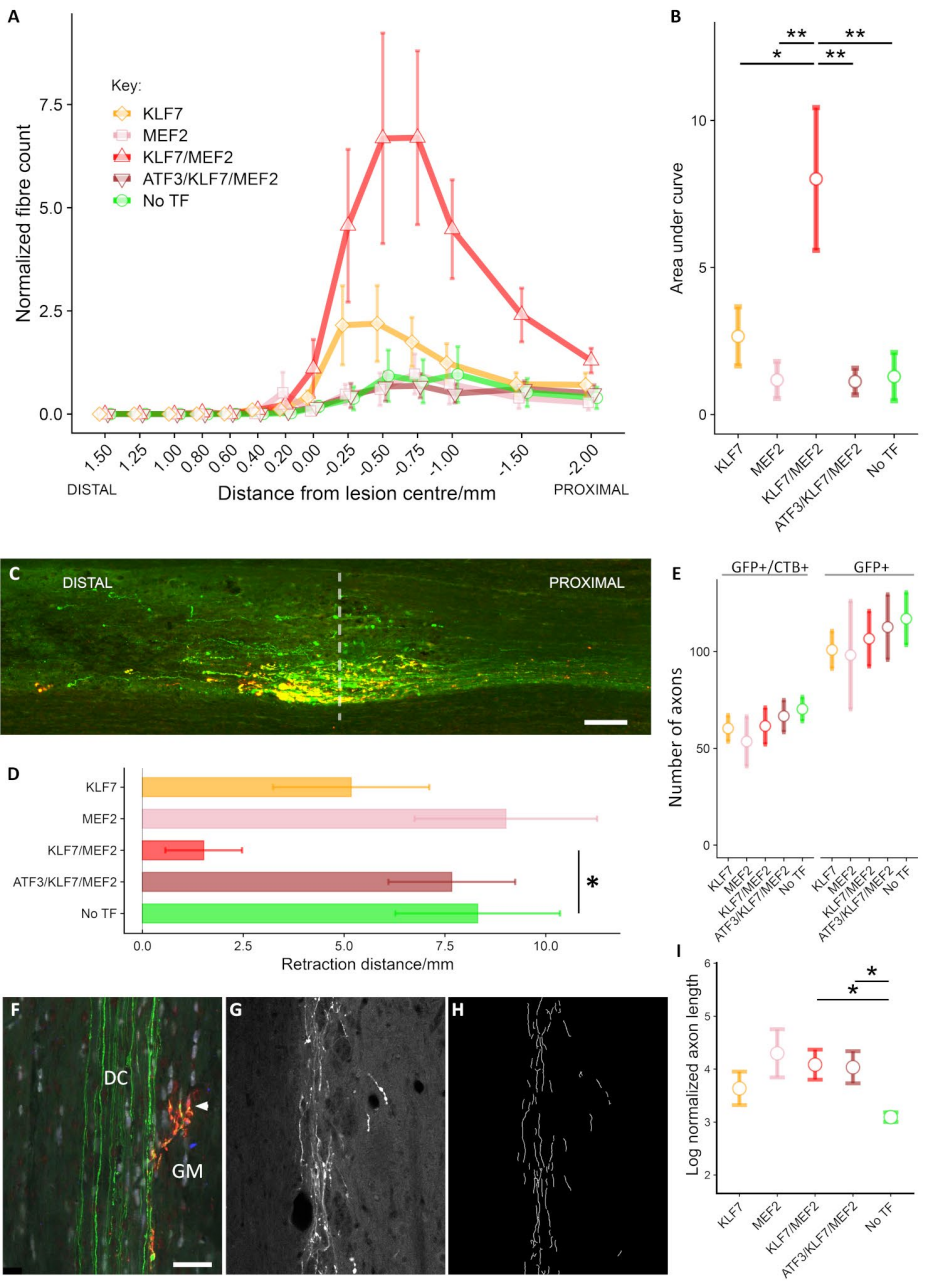
KLF7/MEF2 promotes axon sprouting around the lesion site, prevents retraction and promotes sprouting into proximal spinal cord grey matter **(A)** Quantification of sprouting at the lesion site for all groups. Axon counts at each distance were divided by the number of axons present at -3 mm **(B)** Average total growth (mm) per axon at the lesion site, calculated as the area under the curve in A. KLF7/MEF2 overexpression promoted significantly more sprouting compared with all other groups (linear model, F-test *p* = 0.002, with Tukey’s post-hoc tests). **(C)** In many animals we saw substantial retraction of dorsal column axons after the lesion. Shown here is an example of a cluster of end bulbs of retracting GFP-labelled fibres, over 5 mm caudal to the lesion. The centres of such clusters were used to define the retraction distance (white dashed line). **(D)** Quantification of axon retraction in all groups. 0.0 mm represents the caudal lesion edge defined by GFAP staining. Average axon retraction distance was significantly less in the KLF7/MEF2 group than in the GFP group (*p* < 0.05; linear model with F-test and Dunnett’s post-hoc test with GFP as control). **(E)** Total labelled axon counts of the intact region of the dorsal column, defined as the area caudal to the retracting axons. No significant differences were found between groups in the numbers of CTB/ GFP positive (double-labelled) axons, or of GFP positive axons (right). **(F)** An example of GFP and CTB labelled axons (arrowhead) in the dorsal column (DC) leaving the main tract and entering the grey matter of the spinal cord at T8 in the region of Clarke’s column. (green: GFP, red: CTB). **G**, **H**. Example of tracing of axon collaterals in the thoracic spinal grey matter by the Neuromath software. G shows immunohistochemistry of GFP-positive axons and H shows the traced axons identified by Neuromath. **I**. Log of average traced collateral length, normalised to total GFP positive axon counts in the dorsal column. ATF3/KLF7/MEF2 and KLF7/MEF2 overexpression promoted significantly more sprouting than GFP (linear model with F-test and Dunnett’s post-hoc test with correction for heteroscedasticity; F-test *p* < 0.01). * *p* < 0.05 ** *p* < 0.01 for post-hoc tests. *n* = 8 for No TF, MEF2; *n* = 9 for other groups. Scale bars: C: 200 μm; F: 50 μm (applies to F, G,H)

Transganglionic tracing of injured dorsal column axons was performed using CTB 12 weeks after injury. The lesion site was visualized with GFAP immunolabelling, and the ascending fibres were immunolabelled with GFP and CTB (Fig. 4D-M). In the KLF7/MEF2 group, axons were seen entering the lesion and penetrating as far as just short of the distal border (Fig. 4H and I). In the ATF3/KLF7/MEF2 group, a few axons crossed the proximal lesion border but penetrated only 100–200 μm (Fig. 4J and K). In the remaining groups (MEF2, KLF7, No-TF) no axons penetrated into the lesion. Animals in the KLF7/MEF2 group also appeared to show a greater density of labelled axons just caudal to the lesion. To quantify axon growth here and within the lesion, a grid was laid over images of the lesion and all GFP and CTB labelled axons crossing incremental gridlines were counted. We observed significantly more axonal sprouting at the lesion site from injured transduced dorsal column axons in the KLF7/MEF2 group compared with MEF2-only, ATF3/KLF7/MEF2, or No-TF (*p* < 0.01) and to KLF7-only (*p* < 0.05) (Fig. 5A, B).

In many of the animals, mainly in the groups other than KLF7/MEF2, there were very few or no labelled axons in the dorsal column at the lesion site or up to 4 mm caudal to it, indicating that many of the injured axons had retracted substantially. The number of animals with fewer than 5 axons in this region per group were KLF7: 4/9; MEF2: 6/8; KLF7/MEF2: 1/9; ATF3/KLF7/MEF2: 4/9; No TF: 5/8. To determine the extent of the retraction, spinal cord segments caudal to the lesion site were processed for immunohistochemistry. In these sections, large clusters of retracting axons were observed, tipped by retraction bulbs (Fig. 5C). Retraction distances were measured using the centre of these retraction bulb clusters. In the KLF7/MEF2 group, mean axon retraction distance was significantly less than in the No-TF group (*p* < 0.05) with an average retraction distance of 1.5 mm caudal to the lesion (Fig. 5D) compared with around 8 mm in the No-TF group, MEF2 and ATF3/KLF7/MEF2 groups. Note that all but one animal in the KLF7/MEF2 group had axons close to the lesion site, so retraction was partial. Retraction in the KLF7 group was 5 mm (n.s.) and in the MEF2 and ATF3/KLF7/MEF2 groups was similar to No-TF. CTB and GFP positive axons were quantified in intact dorsal columns caudal to the clusters of retraction bulbs (Fig. 5E) and were similar in number in all groups, indicating a comparable level of transduction, survival and GFP expression between groups.

We observed greater retraction after dorsal column lesion than has previously been reported (e.g [75]). To exclude the possibility that AAV vector-mediated transgene expression was causing excessive retraction we carried out dorsal column lesions on three additional groups; one without viral vector injection, and two with AAV-GFP injection to the L4/L5 DRG with and without a left sciatic nerve conditioning lesion. After 12 weeks, the extent of retraction was similar in all groups (Supp. Fig. S6), indicating that significant axonal retraction occurs after a cervical dorsal column lesion, regardless of intervention. Notably, a conditioning lesion, known to encourage regeneration immediately after dorsal column lesion, does not prevent axonal retraction occurring after 12 weeks. These data indicate that the observed axonal retraction after TF overexpression was not likely to be caused by AAV vector delivery or the co-expression of GFP.

Expression of KLF7/MEF2 in DRG neurons led to increased axon sprouting at the lesion site and less long-distance retraction, but it is unclear how these might lead to improved functional recovery. However, changes in innervation of the spinal grey matter occurred caudal to the lesion could potentially lead to functional improvement since neurons here may be able to relay signals to the brain. For example thoracic spinal cord contains the nucleus of Clarke, known to relay proprioceptive information to the cerebellum. For this reason it is interesting to determine the capacity for growth of axon collaterals of injured neurons in the grey matter, so we looked for evidence of increased sprouting in spinal grey matter caudal to the lesion.

Sections of T8 cord containing dorsal column white matter showed evidence of collaterals leaving the ventral dorsal column and entering the grey matter (Fig. 5F-H). Quantification of the lengths of axon collaterals in the KLF7/MEF2 and ATF3/KLF7/MEF2 treated animals was significantly higher than that of No-TF treated animals (*p* = 0.016 and *p* = 0.035 respectively, Fig. 5I). KLF7-only and MEF2-only also appeared to have on average more collaterals than No-TF but differences were not significant.

### Gene expression profiling of TF-expressing DRG neurons

We next aimed to determine whether overexpression of the TFs leads to induction of the RAG program, or part of it. For this we delivered AAV5 dual vectors where each TF was coupled with a different fluorophore: MEF2 with GFP, KLF7 with mCherry and ATF3 with mito-YFP. Each vector bearing a single TF or the vector combinations KLF7/MEF2 or ATF3/KLF7/MEF2, or the No-TF vector, were injected into L4/L5 DRG. In parallel, another set of animals received a sciatic nerve injury with 1 day or 7 day survival, with fluorescent retrograde labelling of the injured neurons, or no injury. From all animals, fluorescently labelled large diameter neurons (or unlabelled in the uninjured, non-injected controls) were laser dissected and their RNA extracted (80–255 neurons per animal) and processed for RNASeq. This experimental design allowed us to compare the gene expression changes induced in large-diameter DRG neurons by TF overexpression with those induced by axotomy. Of the order of 10^7^ read counts were obtained for all samples.

**Fig. 6:**
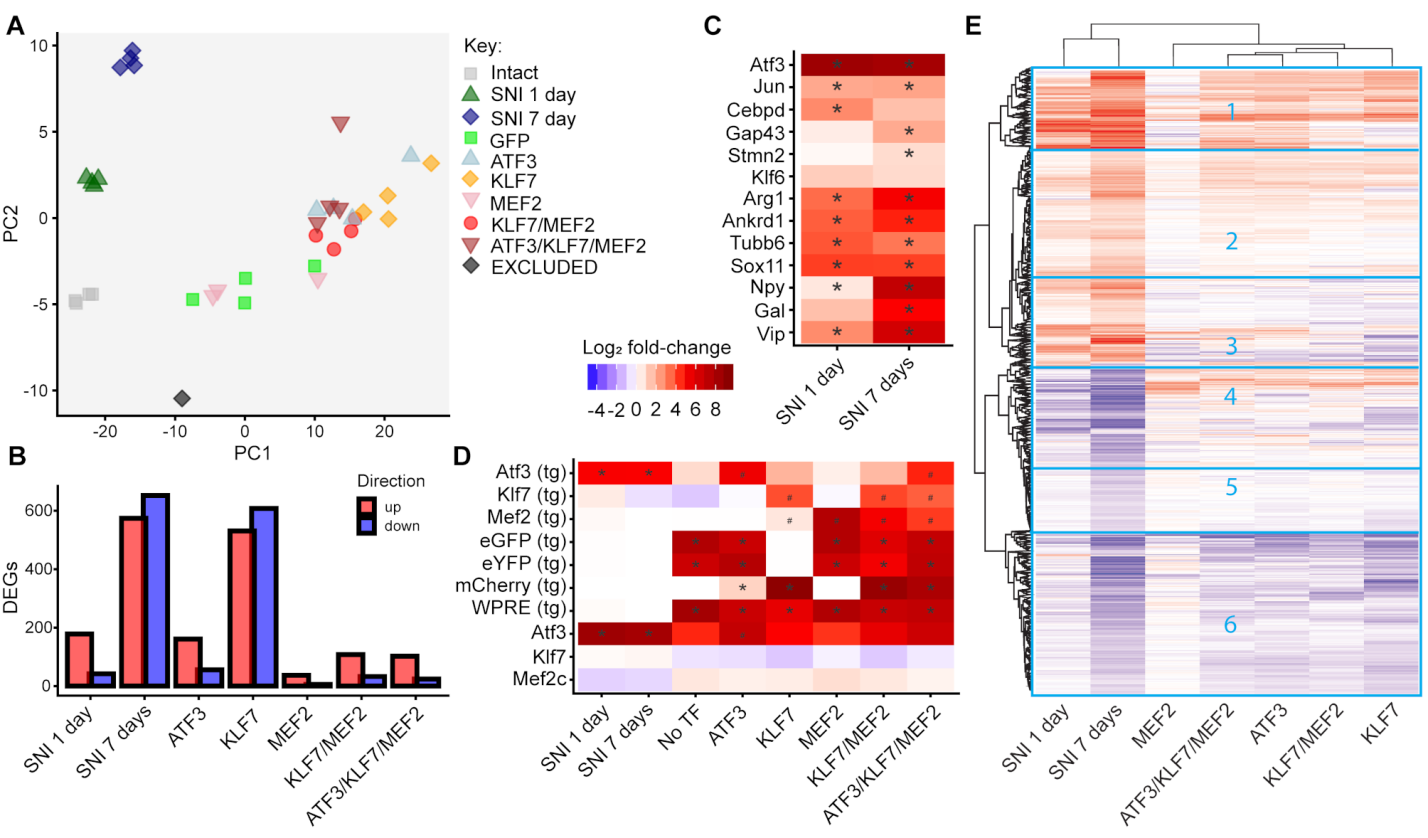
Gene expression changes induced by transcription factor (TF) over-expression, or by axotomy, quantified by RNASeq on laser dissected large-diameter dorsal root ganglion (DRG) neurons. **(A)** Principle component analysis. The first principal component (PC1) appears to reflect changes induced by AAV vector injection and transduction, while the second component (PC2) appears to primarily reflect changes induced by axotomy (i.e. the RAG program). AAV-injection itself does not appear to induce changes in PC2 but TF overexpression does in several groups, suggesting TF expression causes some induction of axotomy-like changes in gene expression. **(B)** Numbers of differentially expressed genes induced by each TF overexpression group. **(C)** Expression of a panel of RAGs following axotomy. With the exception of KLF6, all RAGs were induced by axotomy, showing that RNASeq on laser dissected neurons has good sensitivity. * False Discovery Rate < 0.05. **(D)** Expression of endogenous ATF3, KLF7 and MEF2 and viral-vector-expressed TFs and fluorescent marker genes in nerve injury and TF-overexpression groups. TF and fluorophore expression was detected as expected in all TF over-expressing groups. Note that endogenous and transgene ATF3 have identical coding sequences and so are not well distinguished. Similarly GFP and YFP also have almost indistinguishable sequences. **(E)** Clustered heat map of gene expression log fold-changes for RAGs in DRG neurons overexpressing TFs and days after sciatic nerve injury (SNI). TF-overexpressing groups cluster together in a separate tree to the SNI groups. In the TF groups ATF3 and ATF3/ KLF7/ MEF2 are the most similar to each other, while MEF2 is the least similar to the others. The majority of RAGs display broadly similar patterns of gene expression changes between the SNI at 1 and 7 days and the TF groups, outlined in boxes 1, 2, 5 and 6, with genes being up-regulated in 1 and 2 and down-regulated in 5 and 6. Boxes 3 and 4 contain the smaller group of genes where the SNI groups have opposite expression patterns to the TF groups. Key to log fold changes applies to **B**, **C**, **D** and **E**

Principal component analysis of the 500 most variable genes showed consistent gene expression profiles within groups, although virus-injected groups displayed more variation than naïve and nerve injury groups (Fig. 6A). One sample injected with AAV5-MEF2, was located well apart from the other samples (possibly due to poor sample quality or contamination) and was thus excluded from further analysis. Notably, the MEF2 group was close to the No-TF group suggesting MEF2 had little effect on gene expression.

The non-transduced nerve injury groups appear to be separated from the AAV-injected groups primarily by a difference in Principal Component (PC) 1, while PC2 appears to primarily reflect changes induced by axotomy (i.e. the RAG program). AAV-injection itself does not appear to induce changes in PC2, suggesting AAV injection by itself does not induce an axotomy-like response. However, several TFs or TF combinations do induce changes in PC2 suggesting these factors partially replicate the axotomy-induced RAG expression program.

Differentially expressed genes (DEGs) were determined, comparing expression after nerve injury with uninjured neurons, and comparing neurons of the AAV-TF injected groups with AAV-No-TF. The numbers of DEGs are shown in Fig. 6B. Nerve injury induced increased expression of 177 genes at 1 day and 573 at 7 days. The numbers of DEGs induced by TF over-expression varied considerably, with KLF7 causing the most upregulated genes (528 genes). Curiously, MEF2 induced relatively few DEGs (36 upregulated genes), indicating it had little transcriptional activity in vivo, despite containing the VP16 activation domain. This is consistent with the PCA plot in Fig. 6A. KLF7/MEF2, the most effective combination in promoting regeneration and functional recovery, induced 105 upregulated DEGs and 31 downregulated. All DEGs are listed in Supplemental Data 1.

A panel of 13 well-known RAGs, including ATF3, Jun, Sox11, and Gap43 were all significantly induced by axotomy except for KLF6, validating the axotomy dataset (Fig. 6C). Expression of the expected vector-expressed TFs was clearly present in all appropriate samples (Fig. 6D), validating the experimental approach.

A major aim of this experiment was to determine to what degree TF expression recapitulates axotomy-induced gene expression. We began by hierarchical clustering of expression profiles for all genes that were regulated 1 and 7 days after sciatic nerve injury (i.e. RAGs). Overall, RAGs were more strongly regulated after 7 days than after 1 day. In the TF-expressing groups excluding MEF2, the majority of genes were similarly regulated across the four groups. Considerable similarity to the RAG profile was also apparent with many genes being regulated in a similar direction (Fig. 6E. Boxes 1, 2, 5 and 6). Expression changes induced by MEF2 were minimal compared with the other groups and not similar to the RAG profile or other TF groups.

**Fig. 7:**
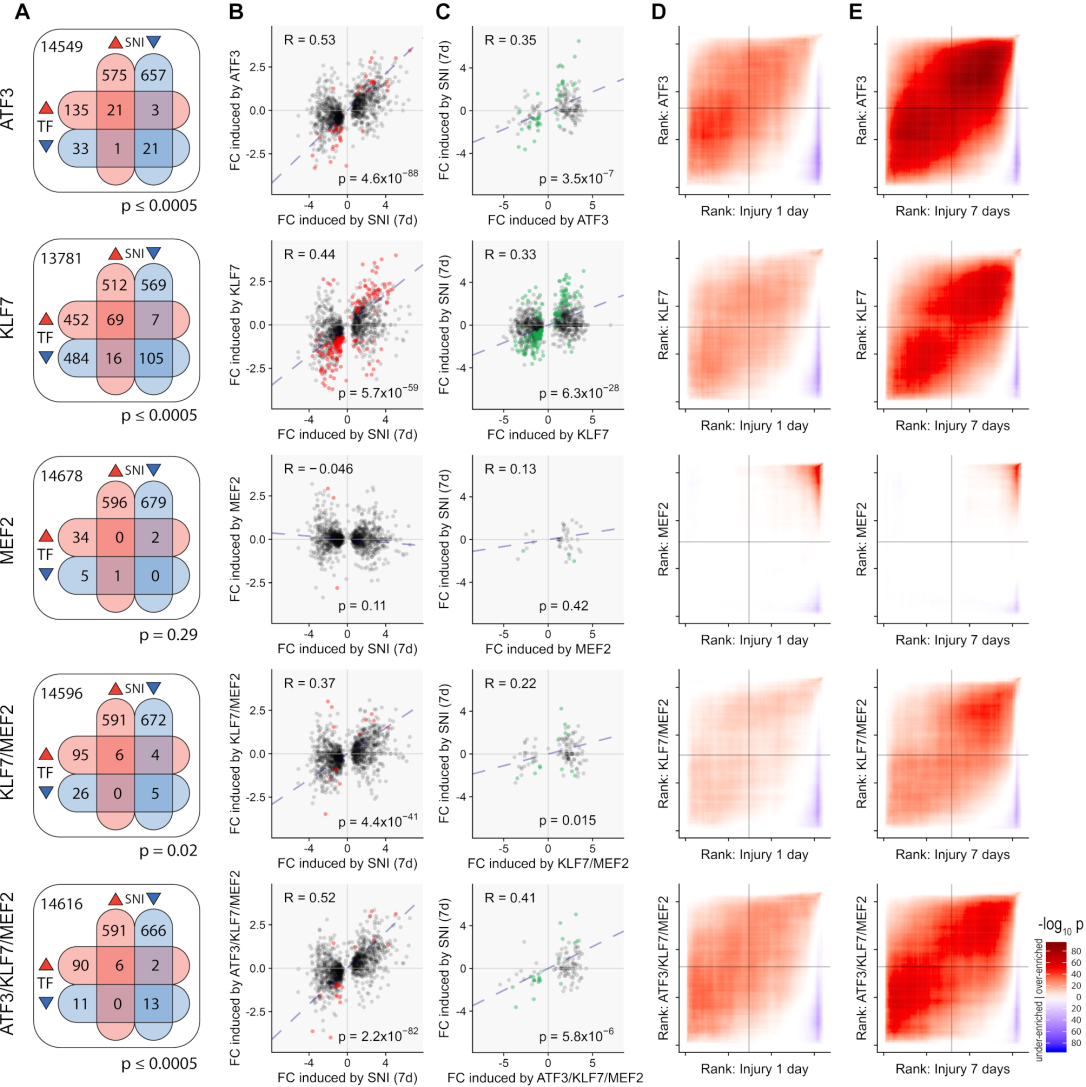
Analysis of concordance in gene expression changes induced by transcription factor (TF) overexpression and axotomy. **(A)** Venn diagrams showing the overlap in differentially expressed genes (DEGs) induced in each direction by TF over-expression and by axotomy. DEGs induced at 1 and 7 days after axotomy were pooled for this analysis. In all cases except MEF2 the overlap was significant (*p*-values given by each diagram; Fisher’s exact test). **(B)** Correlations of log fold-changes (LFC) induced by TF-expression (with respect to the No-TF vector) and by axotomy (with respect to uninjured neurons) in DEGs induced by axotomy. Correlations are significant for all TF groups except MEF2 (Pearson’s correlation test). Red dots indicate genes also significantly altered by TF overexpression. **(C)** Converse analysis to B; correlations of LFC induced by axotomy with LFC induced by TF overexpression for all DEGs induced by each TF combination (*p*-values from Pearson’s correlation test). Green dots indicate genes also significantly altered by axotomy. **D**, **E**. Rank-rank hypergeometric overlap analysis of TF induced gene expression changes and axotomy-induced gene expression changes, for all genes. Significance values of the overlaps in the first *x* and *y* genes of each list are plotted according to the colour key (after multiple testing correction). Highly significant overlaps are found in the ranked gene lists for each TF group except MEF2, with the ranked gene lists for 7 day axotomy, and to a lesser degree, with the ranked gene lists for 1 day axotomy. For all the analyses shown in A-E, ATF3, KLF7 and ATF3/KLF7 show strong concordance of TF induced changes with axotomy induced changes, while KLF7/MEF2 shows slightly less but still highly significant concordance

We next looked for overlap between TF-induced DEGs and a combined list of axotomy-induced DEGs from both time-points after nerve injury (Fig. 7A). ATF3, KLF7, KLF7/MEF2 and ATF3/KL7/MEF2 showed significant overlap in up and down-regulated DEGs (Fisher’s exact test), with KLF7 sharing the most gene expression overlap with the RAGs (174 concordant genes). ATF3 had 42 concordant genes and MEF2 none. Surprisingly, combinatorial expression of TFs resulted in fewer total genes regulated compared with ATF3 or KLF7 alone, and fewer concordant genes (11 for KLF7/MEF2, 19 for ATF3/KLF7/MEF2).

We next examined the correlations in log_2_ gene expression fold-changes after axotomy and after viral vector delivery. In all TF groups except for MEF2-alone, we found significant correlations with the fold-changes of RAGs at 7d post injury (Fig. 7B). These were strongest in the ATF3/KLF7/MEF2 and ATF3 groups, followed by KLF7 and KLF/MEF2. Weaker but still highly significant correlations were also found with 1 day post-injury fold-changes (not shown). These results indicate that almost all the TF expression groups partially induce an axotomy-like response in gene expression when considering RAGs. The strongest correlation was found with ATF3, followed by ATF3/KLF7/MEF2.

We then repeated this analysis on genes significantly induced by each TF (Fig. 7C). Even though most of the genes induced by TF expression were not classified as RAGs (see Fig. 7A), some gene expression changes induced by TF combinations are also induced by axotomy. Fold-change correlations were significant between 7 day sciatic nerve injury and all TF groups except MEF2, and between 1 day sciatic nerve injury and all TF groups except MEF2 and KLF7/MEF2 (not shown).

We also compared the gene expression changes induced by TF overexpression and axotomy using Rank-Rank Hypergeometric Overlap (RRHO) [58], which looks for similarities in gene expression profiles by comparing ranked lists of all genes. Except for MEF2, significant concordance in gene rankings was observed between all TF overexpression groups and RAGs (Fig. 7D, E). Significant overlap is evident along the entire diagonals of the RRHO plots, indicating similarities in the gene rankings is not confined to the most strongly differentially expressed genes but is present when all genes are considered. Concordance was in all cases stronger with the 7 day axotomy group than with the 1 day axotomy group (compare Fig. 7E with 7D), and ATF3 produced the strongest concordance, followed by KLF7, ATF3/KLF7/MEF2 and KLF7/MEF2.

To summarise, with the exception of MEF2 which had little effect on gene expression, all TF groups induced gene expression changes that recapitulate nerve-injury induced changes to varying degrees. Surprisingly, KLF7/MEF2, which produced the most regenerative sprouting and functional recovery, induced gene expression changes with the least similarity to the endogenous RAG program (excluding MEF2).

**Fig. 8:**
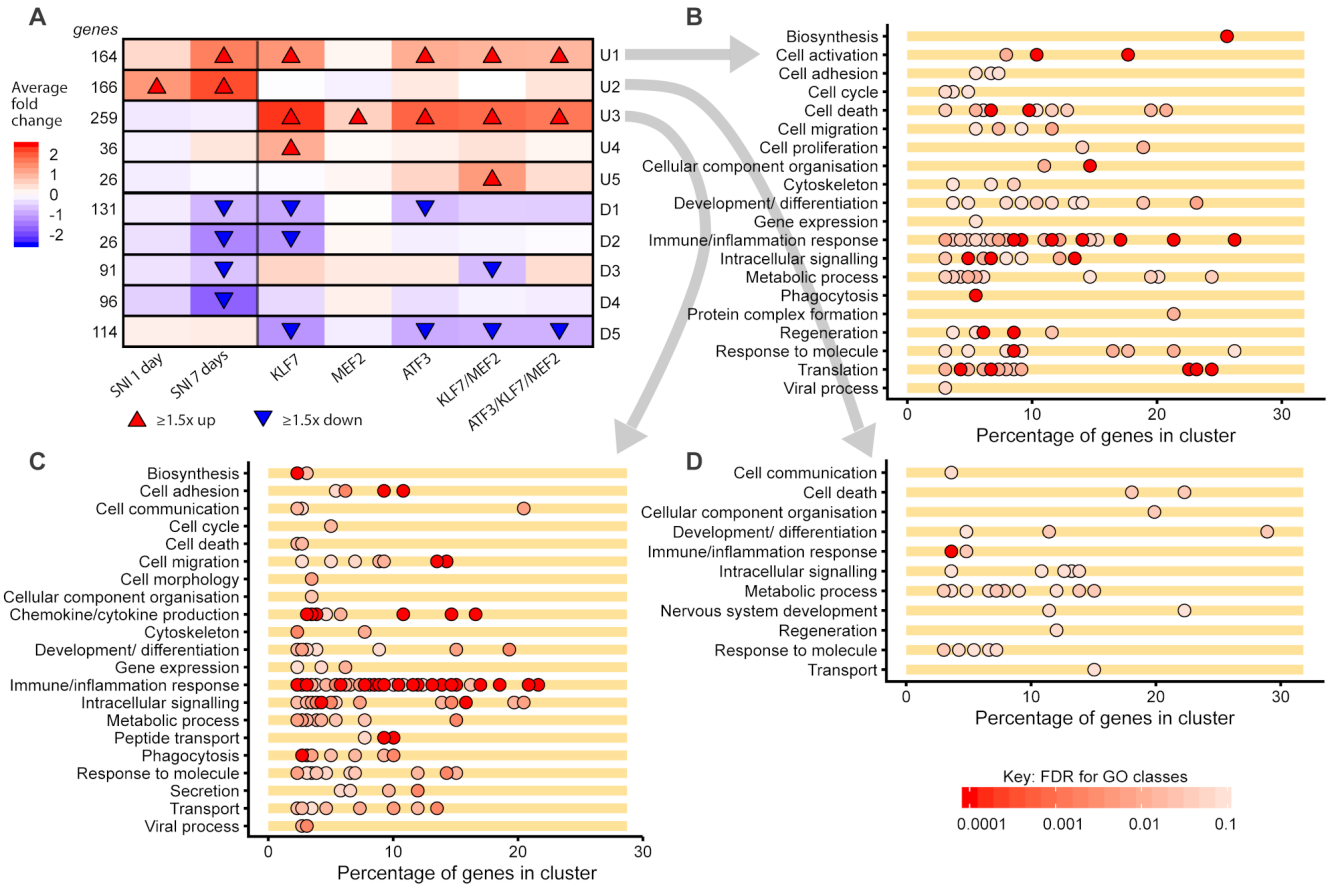
Clustering of gene expression changes induced by transcription factor (TF) overexpression in DRG neurons, and Gene Ontology (GO) over-presentation analysis of three primary clusters. Genes were clustered by Weighted Gene Correlation Network Analysis (WGCNA). **(A)** WGCNA-derived clusters with changes in expression indicated per group for each cluster. Gene expression changes were similar between TF groups (excluding MEF2) and only small clusters of genes specific for different TF groups were identified. Clusters U1, U2 and U3 were the major clusters of genes that were upregulated in TF groups and/or by axotomy. **(B)** U1 contains genes going up after axotomy and in the TF groups excluding MEF2, and contains genes related to biosynthesis, cell death, immune/inflammatory processes, signalling, translation, and regeneration amongst other categories. **(C)** Cluster U3 contain genes only induced by TF expression and contains many genes related to immune and inflammation responses, signalling, cytokine production amongst others. **(D)** Cluster U2 contains genes induced by axotomy and not by TF delivery, i.e. parts of the RAG program missing from the TF-induced responses. Most prominently it contains genes related to metabolic processes, signalling and responses to various molecules. Other clusters are illustrated in Supp. Fig. S7

To further understand the differences and similarities between TF-induced gene expression and the RAG program, we carried out gene expression profile clustering with WGCNA. After merging of similar clusters, a total of 20 clusters remained which were then characterized according to the average fold-change of the genes in each cluster (Fig. 8A). GO over-representation analysis was performed on these clusters, focusing on the Biological Process ontology. Of particular interest were the first three clusters shown in Fig. 8A, U1, U2 and U3. Cluster U1 contains genes which are upregulated by axotomy at 7 days and also by TF delivery for all TF groups except MEF2. These genes represent “real” RAGs induced by TF delivery. Cluster U2 contains genes which are upregulated by axotomy but not by any TF group, and so represent RAGs that are missing from the TF-induced gene expression program. Cluster U3 contains genes upregulated only by TF delivery (all TF groups) and so represent non-RAG TF-induced genes. Many GO classes were over-represented in these clusters, summarized in Fig. 8B, C and D. In Cluster U1 (Fig. 8B), GO classes related to cell activation, translation, development, metabolic processes and intracellular signalling were common, as well as immune/inflammatory classes and cell death. Cluster U2 (‘missing’ RAGs; Fig. 8D) contained fewer GO classes but signalling, metabolic processes and response to extracellular molecules were the most common, and regeneration-linked classes were also present. In cluster U3 (TF-induced non-RAGs; Fig. 8C) numerous classes related to immune and inflammatory processes were present but also many classes relating to intracellular signalling, transport, cell migration and metabolism. GO analysis results for the remaining clusters in Fig. 8A are shown in Supp. Fig. S7. The full lists of over-represented GO classes are given in Supp. Data 2.

The WGCNA analysis gives insight into the patterns of expression induced by TFs, and it is notable that apart from the largely inactive MEF2, all TF groups again (as also seen in Fig, 6E) appear to induce similar gene expression patterns. Clusters U5 and D3 contain genes specifically regulated by KLF7/MEF2 (up in U5–26 genes, down in D3–91 genes), but only one GO class was over-represented in the U5 cluster (‘GO:0009888: tissue development’, with 6 genes). D3 contained genes related to mRNA processing (12 genes) and macromolecule metabolism. These processes may contribute to axon growth although given the relatively few genes involved it seems unlikely that they explain why KLF7/MEF2 specifically should drive increased regenerative sprouting and functional recovery. Thus, while these two classes may be important for axon sprouting induced by KLF7/MEF2, we did not find functional motifs in these gene groups that link clearly to axon growth or connectivity.

**Fig. 9:**
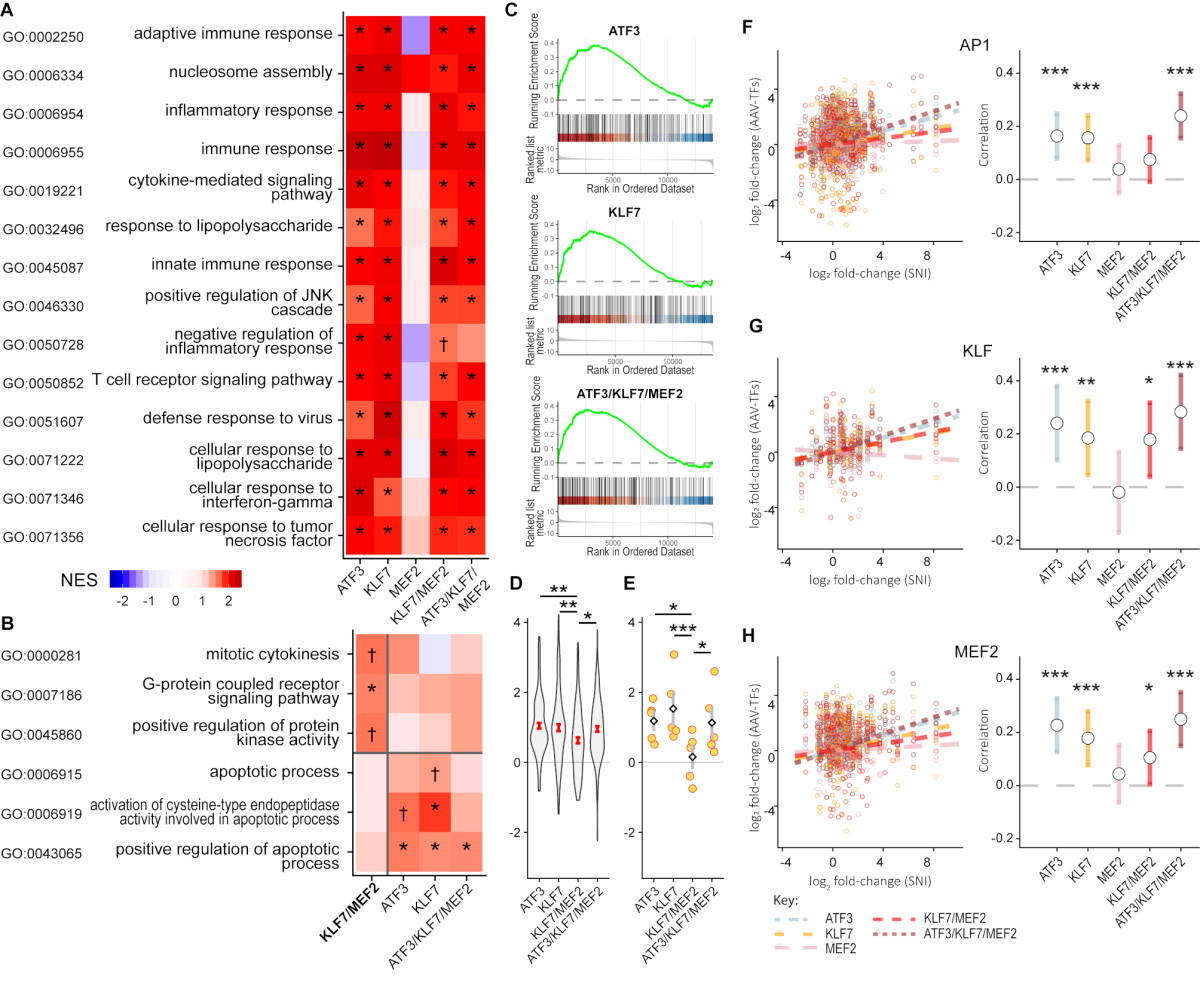
ATF3, KLF7 and ATF3/KLF7/MEF2, but not KLF7/MEF2 induced apoptosis-related genes and specifically caspases. **(A)** Gene set enrichment analysis (GSEA) was carried out on genes upregulated by each TF combination. Shown is a combined plot of the top 10 enriched gene ontology (biological process) classes for ATF3, KLF7, KLF7/MEF2 and ATF3/KLF7/MEF2, showing normalized enrichment scores (NES) and False Discovery Rate (FDR). The top 10 classes were highly similar between these four groups, and consist mainly of classes relating to immune responses and inflammation. **(B)** GO classes specifically over-represented (by GSEA) in genes upregulated by KLF7/MEF2 and not ATF3, KLF7 or ATF3/KLF7/MEF2, and vice versa. The latter three groups induced several classes related to caspase activity and apoptosis. Of note all three of these groups induce the class GO:0043065, positive regulation of apoptosis. **(C)** GSEA enrichment plots for GO:0043065, positive regulation of apoptosis. **(D)** Expression of the pooled leading edge genes for this GO class in the four TF groups excluding MEF2. Expression of this class is significantly lower in the KLF7/MEF2 group than the other three groups (linear mixed model, with Dunnett’s post-hoc test). **(E)** Expression of the caspases Casp1, Casp2, Casp5 and Casp9 (the five caspases in the pooled leading edge gene set shown in **D)** are also lower in KLF7/ MEF2 group (linear mixed model, with Dunnett’s post-hoc test). Yellow dots each represent one caspase, diamonds and grey bars show mean and SEM. **F**-**H**. Analysis of predicted targets for ATF3, KLF7 and MEF2 and correlation of log fold-changes (LFC) for each predicted target set with axotomy-induced LFC. Left panels show scatter plots of LFC induced by TFs vs. LFC induced by sciatic nerve injury (SNI) and lines of best fit, while the right panels show and compare the slopes of the lines of best-fit. Of note genes with predicted AP1 sites in their promoters (putative ATF3 targets) were not significantly induced by KLF7/MEF2, in contrast to ATF3, KLF7 and ATF3/KLF7/MEF2. Key: A,B: † FDR < 0.1; * FDR < 0.05. D-H: *p < 0.05, **p < 0.01, ***p < 0.001, (D, E: linear mixed model, Dunnett’s post-hoc tests with comparison to KLF7/MEF2; F-H: linear mixed model; test for slope being not equal to 0; *n* = 4 animals per group except MEF2 for which *n* = 3)

To gain further insight into the effects of KLF7/MEF2 and the other combinations, we next carried out Gene Set Enrichment Analysis (GSEA) on the gene expression changes induced by TF delivery, testing against the GO (Biological Process) gene sets. The top 10 GO classes with significant enrichment for the four TF groups KLF7, ATF3, KLF7/MEF2 and ATF3/KLF7/MEF2 were highly similar and comprised classes connected to immune response genes (Fig. 9A). We then used GSEA to address the observation that KLF7/MEF2 had positive functional and anatomical effects while ATF3/KLF7/MEF2 and KLF7 alone did not, while these latter two groups appear to better recapitulate the RAG program. To better understand why, we looked for GO classes that were enriched in the KLF7/MEF2-induced gene expression changes and not in KLF7, ATF3, or ATF3/KLF7/MEF2, and vice versa. No GO classes were enriched in MEF2-induced gene expression changes. KLF7/MEF2-specific enriched classes were found for mitotic cytokinesis, G-protein coupled receptor signalling pathway, and positive regulation of protein kinase activity (Fig. 9B). This suggests increases in GPCR signalling pathways and kinase activity in the KLF7/MEF2 group, which could contribute to regeneration.

Conversely, a number of classes related to signalling and transcription were enriched in the KLF7, ATF3, or ATF3/KLF7/MEF2 groups, and notably, also several classes related to apoptosis, none of which were enriched for KLF7/MEF2 (Fig. 9B). In particular, the class GO:0043065 (‘positive regulation of apoptotic process’), was enriched in all three of the former groups and not in KLF7/MEF2. GSEA enrichment plots for this GO class are shown in Fig. 9C. Mature neurons are quite resistant to apoptosis [76, 77] but the apoptosis machinery, and in particular caspases, is involved in axon retraction [78–80]. The enrichment of apoptosis-related classes in KLF7, ATF3 and ATF3/KLF7/MEF2 groups but not in KLF7/MEF2 could therefore be a reason why the former groups were less effective in promoting axon growth. GSEA results include a ‘leading edge’, which is the set of genes most responsible for the enrichment. We looked at TF induced fold-changes in gene expression of the combined set of leading-edge genes for class GO:0043065 from the KLF7, ATF3, and ATF3/KLF7/MEF2 groups. Expression of the combined leading-edge set was significantly lower in the KLF7/MEF2 group than in the other three groups (Fig. 9D). The caspases Casp1, Casp2, Casp3, Casp8 and Casp9, are all members of the combined leading edge gene set. These caspases are also lower expressed in the KLF/MEF2 group than in the other groups (Fig. 9E).

The transcription factor binding site analysis which resulted in the nine factors chosen for in vitro screening (as shown in Fig. 1) also yields sets of predicted target genes. We next examined if the predicted targets of ATF3, KLF7 or MEF2 were upregulated by the TF combinations delivered. The predicted targets were converted to rat homologues and are listed in Supp. Table S4. Correlation plots of TF-induced fold-changes versus fold changes induced by mouse facial nerve injury, along with estimates of the magnitudes of the slopes of these graphs are shown in Fig. 9F, G, H. Predicted target genes of ATF3 (genes whose promoters contain AP1 sites) were significantly induced by ATF3, KLF7 and ATF3/KLF7/MEF2, but not KLF7/MEF2 (Fig. 9F). Genes with promoters containing KLF and MEF2 sites showed a similar pattern, with KLF7/MEF2 showing less induction of these predicted target genes than the other ATF3 and KLF7 containing groups (Fig. 9G, H). This suggests that, in general, induction of these target gene sets correlates with the overall strength of RAG induction and not specifically with expression of the TF associated with each predicted target set. A partial exception however is that KLF7/MEF2, which does not contain an AP1-binding TF, fails to induce predicted targets of AP1.

In summary, the TF combination of KLF7/MEF2 was the most effective in promoting axon sprouting and functional recovery in vivo, and gene expression profiling data suggests its superiority may be due to the partial induction of the RAG program combined with the lack of induction of apoptosis machinery which occurs in other combinations.

## Discussion

In this work we explored the use of transcription factor combinations to artificially activate the axon regeneration program in DRG neurons without the use of a conditioning lesion. Analysis of the promoters of the RAG program in mouse facial motor neurons led to identification of a set of TFs with high likelihood to be pro-regeneration, and we systematically screened these factors to identify the most potent combinations of factors for promoting axon growth.

In vitro, the combination ATF3/KLF7/MEF2 was highly effective at promoting axon growth. Surprisingly, in vivo this combination failed to promote regeneration or functional recovery. KLF7/MEF2 on the other hand induced multiple positive effects, including sprouting into the lesion site and into proximal grey matter, prevention of axonal retraction and improvement in functional recovery. Lastly, analysis of the gene expression changes induced by overexpression of the various TF combinations indicated that KLF7/MEF2 was not the most potent at recapitulating the axotomy-induced gene expression program, and ATF3, KLF7 and ATF3/KLF7/MEF2 were all superior in this regard.

The GO classes upregulated by the TF groups depicted in Fig. 8 suggest a number of mechanisms which may be favourable to axonal sprouting. Notably a large number of genes in cluster U1 are linked to translation and biosynthesis. In particular, local translation has been linked to successful regeneration. Along with classes related to cell activation and metabolism these are likely to be important for the increased capacity to synthesize axonal material necessary during axon growth. GO-classes related to cell migration, cell adhesion and development/differentiation in clusters U1 and U3 may also contain genes directly related to axon growth. The most commonly occurring categories in clusters U1 and U3 were connected to inflammation and immune responses. Inflammatory cytokines have a well-established effect in promoting regeneration and T-cells have a role in the survival of injured neurons but can also be inhibitory to regeneration. Modulation of immune processes may therefore be an important component of the TF-induced RAG programs [81–83].

Previous efforts to promote axon regeneration with TFs targeting DRG neurons have resulted in a similar degree of sprouting at the lesion site and also reduced axonal retraction, but not functional improvement. Gao et al. over-expressed a CREB VP16 construct in DRG neurons and some axon sprouting as far as the lesion centre was seen [10]. Overexpression of STAT3C in DRG neurons with an AAV vector resulted in increased initial sprouting of cut axons in the first 2–4 days but no sustained growth and no growth into the lesion site was shown [9]. Over-expression of ATF3 in DRG neurons failed to promote regeneration into a dorsal column lesion when constitutively expressed in transgenic mice [19] or when delivered by AAV [23] and combined expression of ATF3, JUN, STAT3C and SMAD1-EVE also failed to promote regeneration in this model [23]. In several cases over-expression of TFs in DRG neurons leads to reduced axonal retraction, and this was seen with SOX11 [16], KLF7-VP16 [75] and MYC [84] with some fibres entering the lesion in the last case. The current study is the first where TF-delivery to DRG neurons has been shown to lead to functional improvement in a spinal cord injury model. More success has been attained in the corticospinal tract, where over-expression of KLF7-VP16 [15], SOX11 [16] or KLF6 [17] were found to lead to increased axon growth after spinal cord injury. Expressing STAT3 led to increased sprouting across the midline in this model [85].

The in vitro and in vivo phases of this study yielded somewhat differing results, and the question arises why. The gene expression data generated by RNAseq on fluorescently labelled laser-dissected DRG neurons goes some way to explaining the differences. One important finding is that MEF2 by itself appeared to have little transcriptional activity in vivo. This is somewhat surprising since the construct we used contains the VP16 transactivation domain, which should provide constitutive transcriptional activity. However, the inactivity of MEF2 may be connected to the mechanisms by which MEF2 activity is controlled. Among other mechanisms, MEF2 activity is regulated by Histone Deactylases (HDACs) [86], which bind directly to MEF2. HDACs repress gene expression by promoting nucleosome stability and heterochromatin formation. VP16 acts by recruiting histone acetyl transferases (HATs), and so the modes of action of VP16 and HDACs are directly opposed; it may be that HDAC activity is simply more potent than that of VP16. In this case, MEF2 would function largely as a transcriptional repressor in our experiments. Another possibility is that MEF2-VP16 fails to displace endogenous MEF2 bound to the DNA. MEF2-family factors may have very low turnover of the DNA-bound molecules, since their activity is regulated mainly by co-factor activity and not by DNA binding. In any case, the inactivity of MEF2 is very likely a factor in the discrepancy between the in vitro and the in vivo experiments.

MEF2 family factors have not previously been implicated in the gene expression response to axotomy, but they are well placed to do so. The known targets of MEF2 include Jun, and predicted targets identified here also include ATF3 and KLF7 (see Supp. Table S4). Export of HDAC5 from the nucleus is observed after axotomy [87], suggesting release of HDAC inhibition of targets is a key step in the activation of the RAG program. It is possible that MEF2 factors directly upregulate these RAG TFs following the release of HDAC inhibition.

In our experiments MEF2 appears to have had a damping effect on gene expression induced by KLF7, which, surprisingly, was beneficial. The gene expression data revealed the induction of apoptosis-related genes in all TF groups except MEF2 and KLF7/MEF2, most notably the upregulation of caspases 1,2,5,8 and 9. This suggests that MEF2 may act to partially repress caspase expression in our experiments. In mature neurons, apoptosis is repressed to a large degree [76, 77], and caspase activity instead induces axon retraction [78–80]. It is notable that the addition of ATF3 to the pairing of KLF7 and MEF2 causes increased expression of apoptosis-related genes, including caspases, and abolishes the pro-regenerative effects of this pair. ATF3 by itself also induces this category of genes, and it is known that it does not induce a growth response at a dorsal column lesion site [19, 23]. The caspase-induced retraction pathway is initiated in response to neurotrophin withdrawal [79]. In the conditioning lesion model, not only is RAG expression induced by the axotomy, but the injured neurons also receive additional neurotrophic support from the peripheral nerve, since Schwann cells at the injury site upregulate neurotrophin expression. This may suppress neuronal caspase expression and activity. However, in the experiments carried out here the cell body does not receive this additional neurotrophic support. In this case, despite the large-scale induction of the RAG program by ATF3, KLF7 and ATF3/KLF7/MEF2, we hypothesize that the induction of caspase expression and lack of neurotrophic signalling tips the balance towards retraction rather than regeneration.

Interestingly, the ATF3, KLF7 and ATF3/KLF7/MEF2 groups all showed significant induction of predicted AP1 target genes whereas with KLF7/MEF2 this was absent. This is logical for the ATF3 containing combinations, since ATF3 can bind AP1 sites when dimerized with Jun [88]. AP1 activity in neurons is known to promote expression of apoptosis-linked genes [89].

The approach we have taken here has certain limitations. To begin with, we examined the RAG program in mouse facial motor neurons to determine candidate TFs, whereas the in vivo model in which we tested the chosen factors was the rat DRG. Differences in transcriptional control mechanisms of RAGs between rat and mouse are likely to be minimal, however motor neurons and sensory neurons may have subtle differences in their transcriptional programs. A second limitation in the methodology is the use of F11 cells to screen for effects on neurite outgrowth. While useful, because they show sensitivity to TF overexpression, the assay involves inducing the growth of new axons, akin to developmental axon growth, rather than axotomy-induced regeneration. This limitation ultimately manifested itself in that it failed to properly capture one important element of the transcriptional state of DRG neurons, namely the constitutive repression of MEF2 activity that we observed in vivo. The MEF2C-VP16 construct was transcriptionally highly active in F11 cells (data not shown).

Another limitation of our study is that in our in vivo experiments we used only female rats. Some sex differences have been found in recovery and spinal cord tissue preservation in contusion and compression models of spinal cord injury, with females performing better [90, 91]. Neuron intrinsic responses to axotomy were found to be similar between sexes in mice in terms of RAG expression, although differences in which genes were regulated were found [92]. Peripheral nerve regeneration was found to proceed slightly faster in males in rats [93] but faster in females in mice [94]. Thus while the fundamental mechanisms driving regeneration should function similarly in males and females, in our model there may be sex differences in the effects of TF overexpression, particularly with regard to functional recovery.

In general studies testing neuron intrinsic approaches to promote regeneration have used partial transection or dorsal column crush injuries to sever specific spinal tracts (reviewed in [95]) and similarly, we used a dorsal column transection. It is interesting to consider whether such an approach would work in clinically more relevant contusion models of spinal cord injury. Such injuries have larger and less well-defined lesions, usually with secondary lesion enlargement. Successful treatment of such injuries could require a combination of treatments that provide a suitable substrate for growth at the lesion site, counter the inhibitory nature of the CNS, and also activate neuron intrinsic mechanisms. The approach taken here could thus form one component of such a strategy for these more difficult lesions.

The use of laser dissection microscopy combined with RNASeq to determine the effects of TF delivery provides useful insights into the efficacy of our approach, as well as the complexity of transcriptional regulation and the difficulties that arise in manipulating the transcription state of injured neurons to boost regeneration. One interesting finding is that KLF7 and ATF3 appear to both stimulate large parts of the RAG program with rather similar profiles, suggesting these TFs have a number of target genes in common.

Analysis of the gene expression data suggests that MEF2-VP16 has little transcriptional activity in DRG neurons in the absence of endogenous injury signalling, and also that induction of apoptosis machinery alongside RAGs correlates with failure to induce axonal growth. This in turn suggests that a lack of neurotrophic support may inhibit axon regeneration. Therefore, the next steps in this approach would be to interfere with HDAC inhibition of MEF2 and to provide additional neurotrophic support alongside TF overexpression. An interesting experiment to test these ideas would be to repeat the overexpression of the TF combinations tested here in combination with a conditioning lesion. This would have the advantage that peripheral neurotrophic signalling would be present and the initial signals leading to HDAC disinhibition would also be present. The overexpression of TFs could then be expected to lead to a more sustained RAG program and longer distance regeneration than that induced by conditioning lesion alone. Lastly, it may be that additional TFs are needed to fully reproduce the RAG program induced by axotomy, and it is also possible that knockdown of certain TFs might be necessary to fully recapitulate the regenerative state. However, transcriptional re-programming with TFs is widely used and predominantly utilises over-expression rather than knockdown [27]. Furthermore our gene expression profiling data indicate that TF overexpression not only causes upregulation of genes but also leads to down-regulation of many genes, including genes suppressed after axotomy, suggesting that TF knockdown may not be essential.

Boosting the intrinsic neuronal regeneration potential by manipulating transcription factor activity remains a promising approach to promote regeneration. Other approaches to boosting the neuronal regenerative capacity have also had success, most notably PTEN knockout in corticospinal neurons and retinal ganglion cells [96, 97]. Expression of a growth-promoting integrin along with a co-activator also promoted axon growth into the CNS and an upregulation of RAGs [98], although this has not been shown to be effective in a spinal cord lesion yet. In principle the TF approach should allow a powerful and sustained regenerative response in injured neurons for CNS repair, even if the complexity of transcription regulation in this system renders this challenging.

## Conclusions

We have identified a TF combination (KLF7/MEF2) which activates significant RAG expression in DRG neurons, and promotes axonal sprouting and functional recovery in vivo in the dorsal column injury model. This combination and the others tested here provide a basis for further development to fully activate the regenerative gene expression program and induce long-distance axonal regeneration beyond a spinal cord lesion.

## Electronic supplementary material

Below is the link to the electronic supplementary material.

Supplementary Material 1

Supplementary Material 2

Supplementary Material 3

Supplementary Material 4

## Data Availability

The RNASeq dataset is available at NCBI GEO, accession number GSE270874. All other data generated or analysed during this study are included in this published article and its supplementary information files.

## Abbreviations

AAV: Adeno-associated virus
CL: Conditioning lesion
CNS: Central nervous system
DCL: Dorsal column lesion
DEG: Differentially expressed gene
DRG: Dorsal root ganglion
FDR: False discovery rate
FMN: Facial motor nucleus
GO: Gene ontology
GSEA: Gene set enrichment analysis
IHC: Immunohistochemistry
LFC: Log fold-change
NES: Normalized enrichment score
ORF: Open reading frame
PC: Principal component
RAG: Regeneration-associated gene
SNI: Sciatic nerve injury
TF: Transcription factor
WGCNA: Weighted gene correlation network analysis
