# Supplementary material for: The transcription factor combination MEF2 and KLF7 promotes axonal sprouting in the injured spinal cord with functional improvement and regeneration-associated gene expression
Source: Mol Neurodegener. 2025 Feb 8;20:18. doi: 10.1186/s13024-025-00805-4 (PMC11807332; doi:10.1186/s13024-025-00805-4)
Supplement: Supplementary file 3 — Supplementary Material 3 [file 13024_2025_805_MOESM3_ESM.docx]

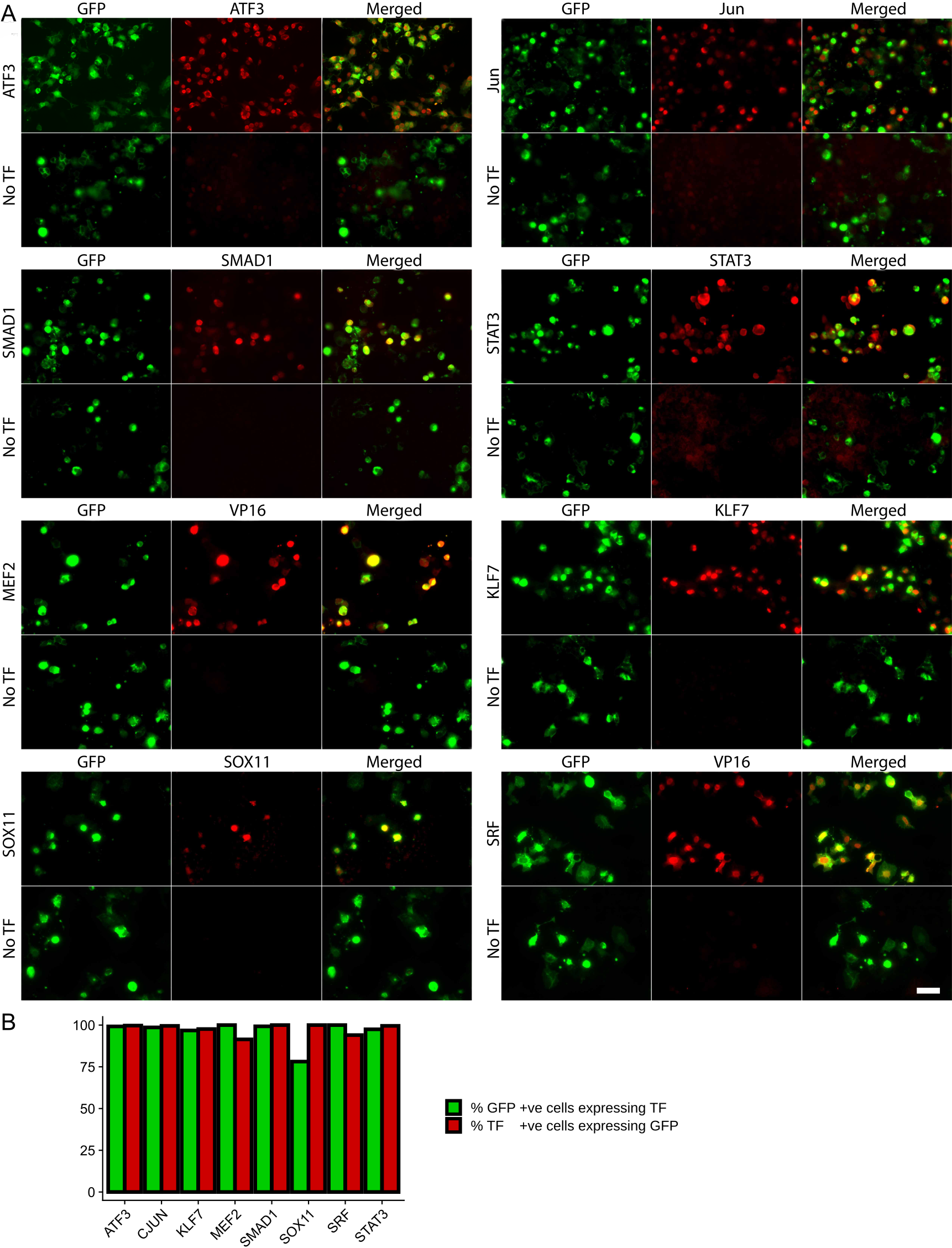
**Fig. S1.** **Overexpression of transcription factors (TFs) in HEK 293T cells using AAV dual promoter plasmids.** A. HEK 293T cells were transfected with AAV dual promoter constructs expressing both a TF and GFP (top row of each panel) or the control ‘No TF’ vector that expresses GFP only (bottom row of each panel). Cells were then fixed and immunostained for GFP and the TF in question. Labels on the left indicate the transfected plasmid and the labels along the top indicate the antibodies visualized. Each plasmid expresses GFP and its TF as expected. Scale bar 50μm. B. Quantification of co-expression of each TF and GFP in HEK cells. In most cases co-expression rates are over 90%. The lowest rate of TF expression in GFP+ cells was for SOX11 at 78%, but this is most likely due to the antibody being of lower quality. Since all TFs were expressed from the same backbone construct we expect expression efficacy to be similar in all cases.


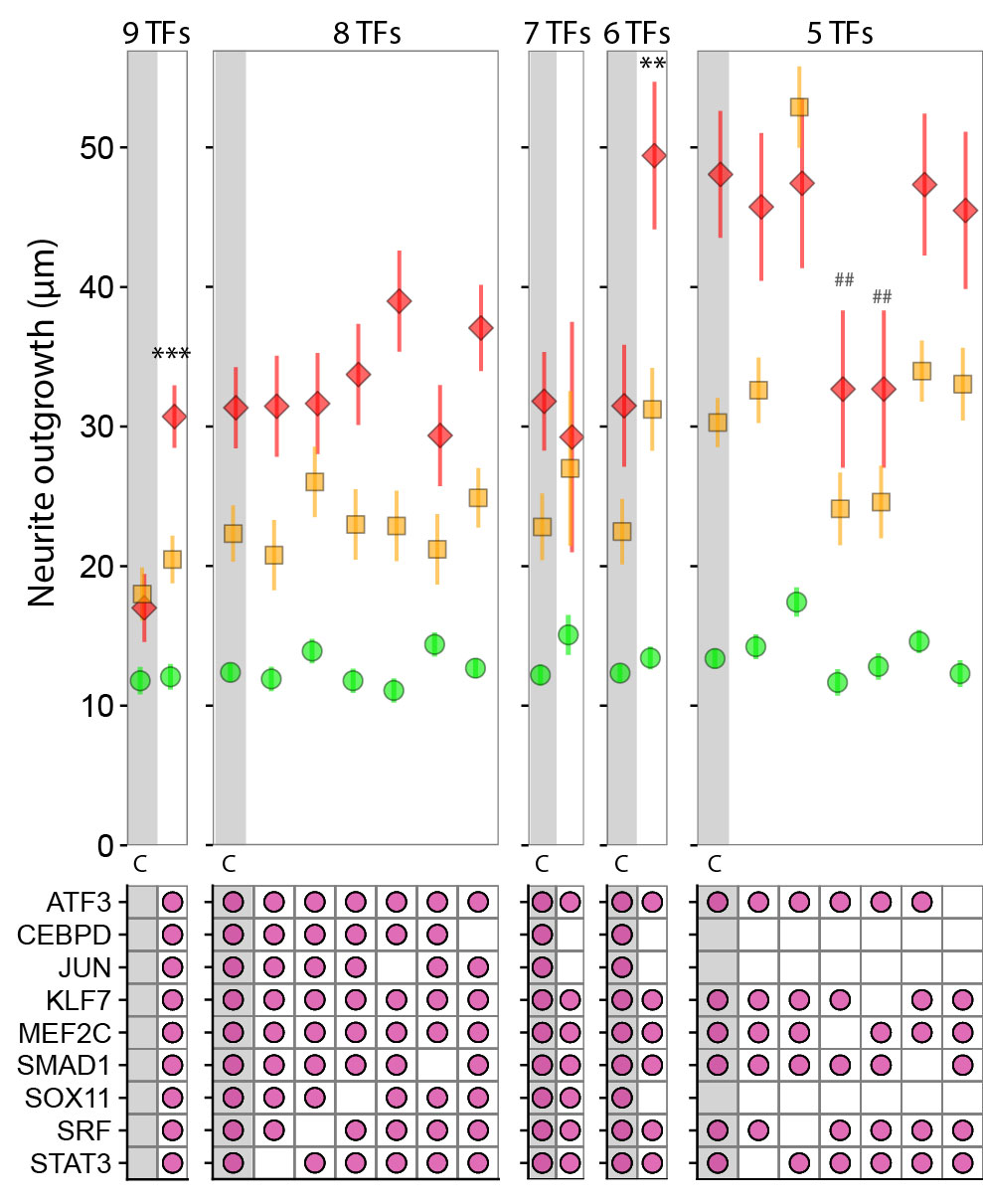


**Fig. S2.** Screening of combinations of transcription factors in F11 cells. Here a subtractive screen was performed beginning with all 9 factors and iterative removal of selected factors. 9 TFs: All 9 factors co-transfected together induced greater outgrowth than the no TF condition, although this was considerably less than that induced by the triple combination ATF3/KLF7/MEF2 (see Fig. 2). 8 TFs: Individual TFs (all excluding ATF3 KLF7 and MEF2) were removed; CEBPD or JUN caused a non-significant increase in outgrowth. 7 TFs: Combined removal of CEBPD and JUN had little effect on outgrowth. 6 TFs Combined removal of CEBPD, JUN and SOX11 had caused a significant increase in outgrowth. 5 TFs Removal of each of the remaining factors did not cause further increases in outgrowth and in fact removal of KLF7 or MEF2 led to a significant drop-off in neurite outgrowth. **, ## p<0.01; ***p<001. Stars indicate a significant increase with respect to the control condition, hashes indicate a significant decrease. (Linear mixed model with Dunnett’s post-hoc tests, n≥4)


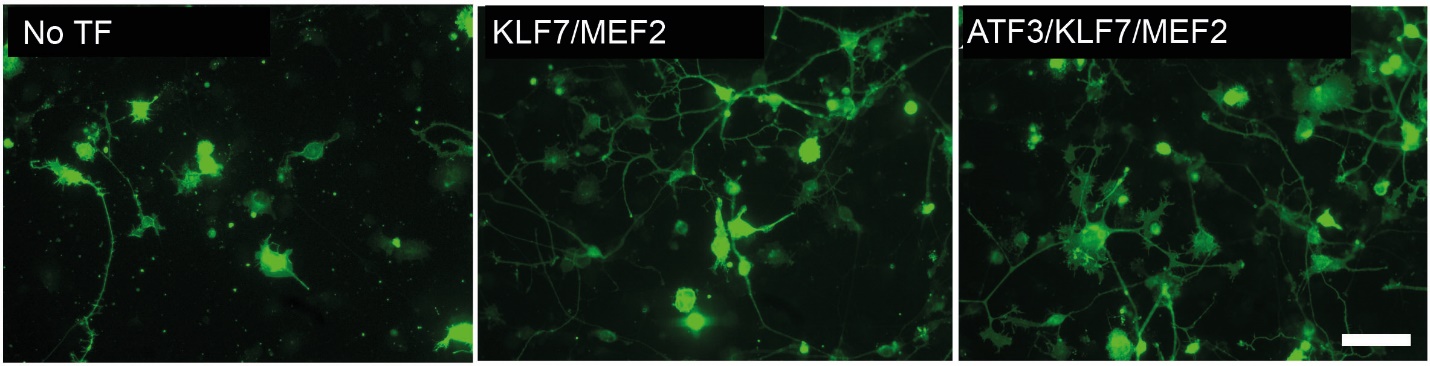


**Fig. S3. F11 outgrowth at 72 hours in the highest performing TF combinations.** Example images of neurite outgrowth after 72 hours in F11 cells transfected with GFP only (No-TF plasmid), KLF7/MEF2 or ATF3/KLF7/MEF2, as used for quantification with the Cellomics ArrayScan HCS Reader. Scale bar 50 μm.


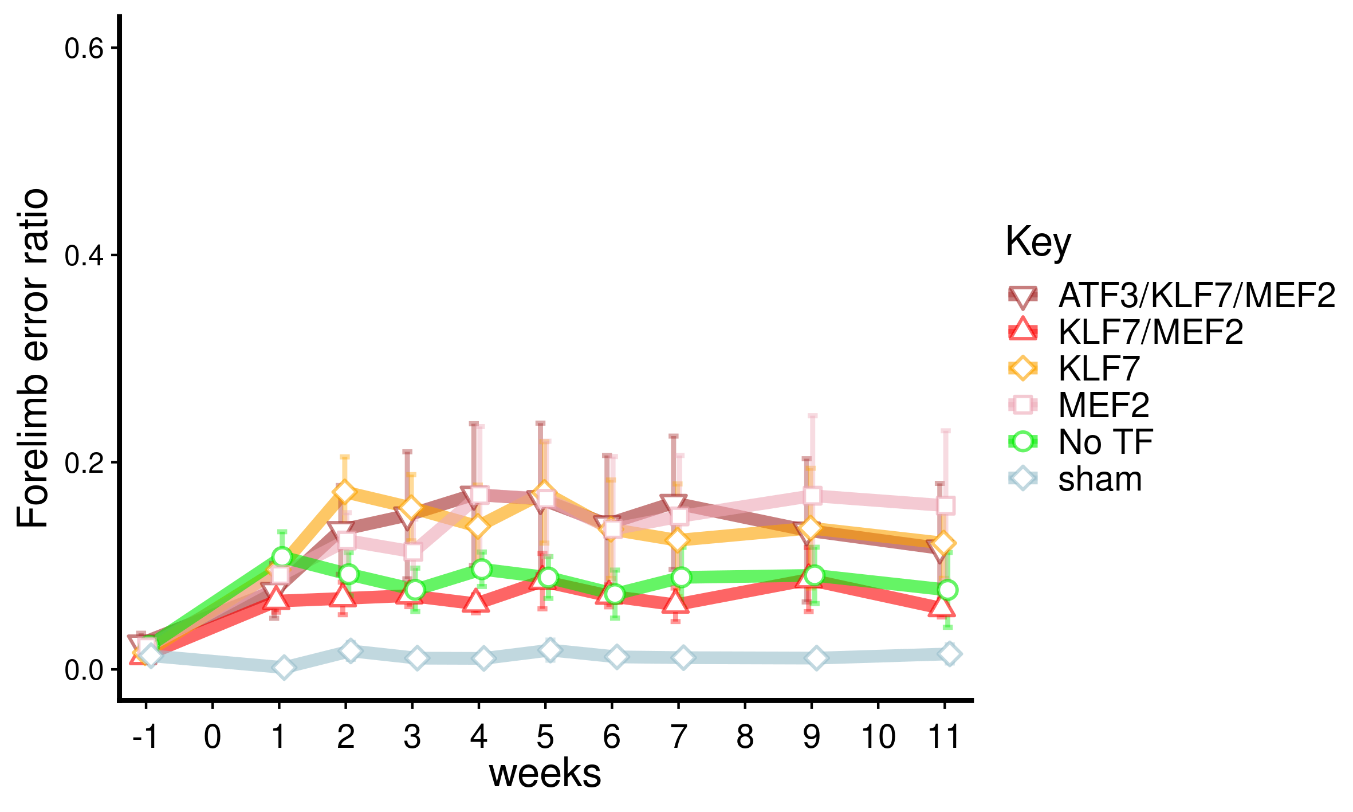


**Fig. S4. Error rates for forelimbs on the horizontal ladder.** Forelimb slips and misses were scored after the cervical dorsal column injuries. Forelimb function is affected by the injury to a minor degree but remains stable across the period. No significant differences were found between any TF group and the ‘No TF’ group, although the sham and ‘No TF’ groups were different (p<0.001; binomial linear mixed models).


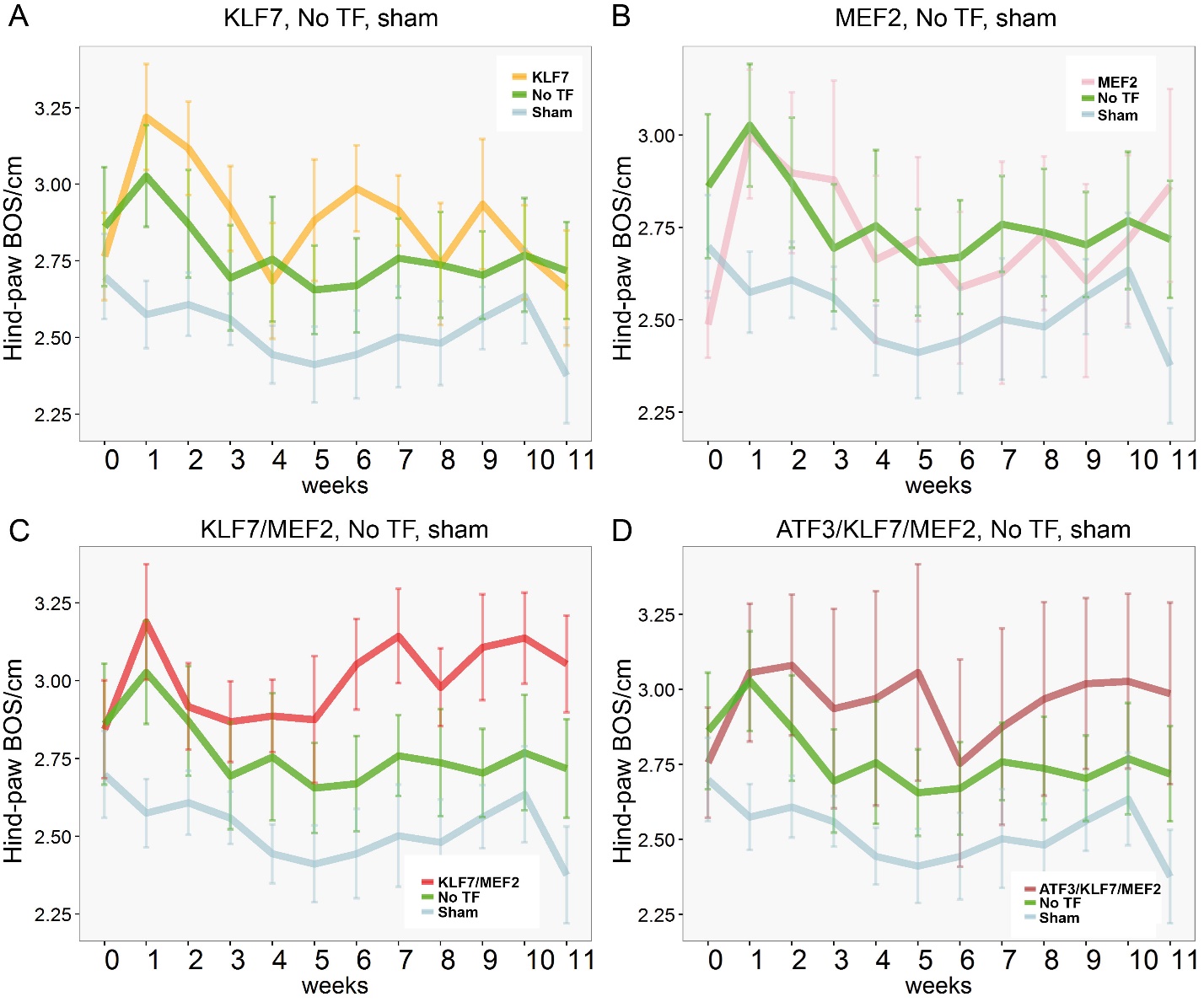


**Fig. S5. Hind paw width following dorsal column lesion**. Animals’ paws were filmed while crossing a walkway and subsequently analysed with semi-automated gait-analysis software (CatWalk XT). Plotted above is the hind-paw ‘Base of Support’ (BOS) measurement (side-to-side distance between the hind paws) in transcription-factor treated animal groups, compared to the No-TF-treated controls and sham-operated animals. (A) KLF7. (B) MEF2C. (C) KLF7/MEF2C. (D) ATF3/KLF7/MEF2C. Animals of the KLF7/MEF2C and ATF3/KLF7/MEF2C appear to show a trend towards a wider base of support after dorsal column lesion, increasing over time. No significant differences were found between groups (linear mixed model; n=8 for No TF and MEF2 groups, n=9 for other groups).


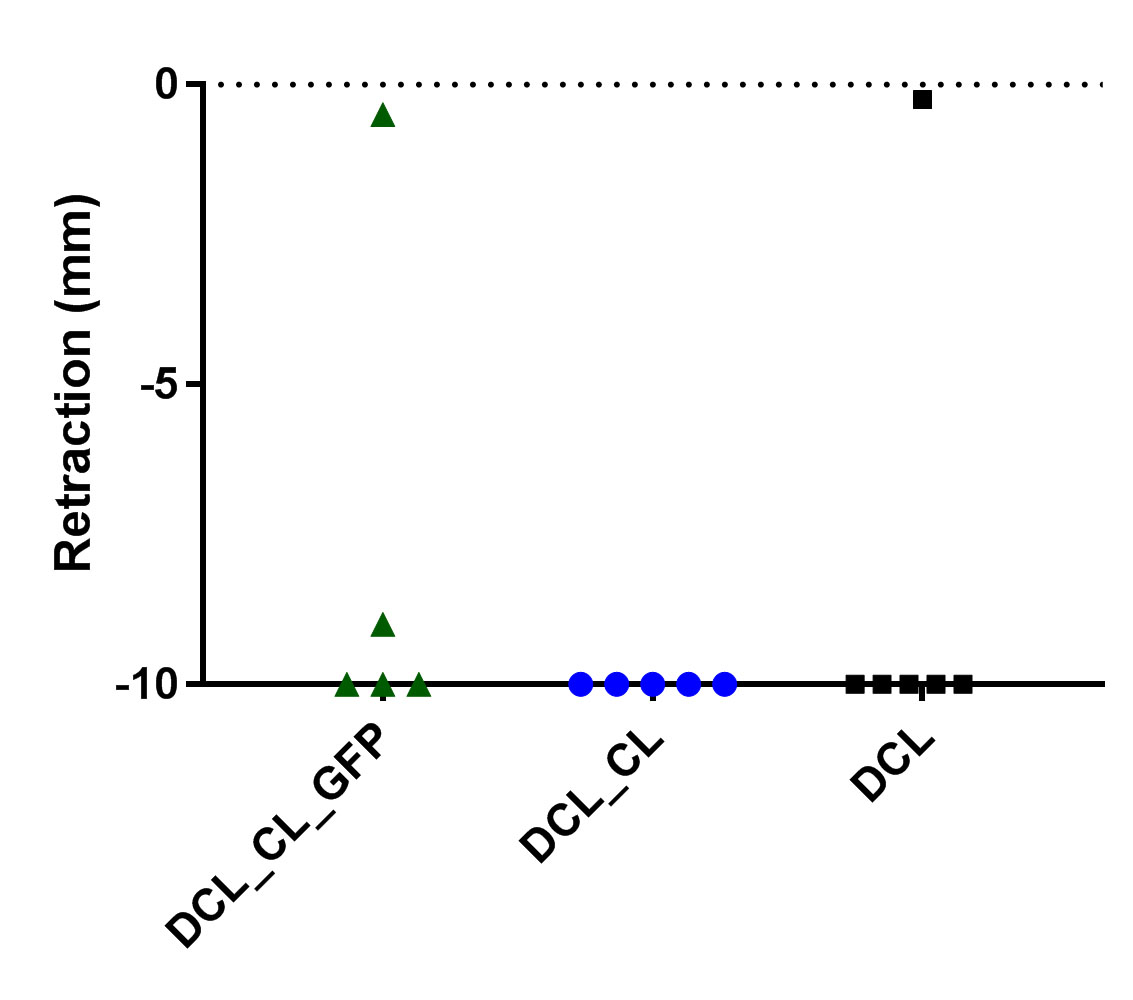


**Fig. S6. Peripheral conditioning lesion does not prevent retraction 12 weeks following dorsal column lesion.** Quantification of axonal retraction after a C4 dorsal column transection in conjunction with a conditioning lesion of the sciatic nerve one week prior to the dorsal column lesion. This was quantified both with and without GFP overexpression. Retraction was also quantified after a dorsal column lesion with no conditioning lesion and no GFP overexpression. **DCL**: C4 dorsal column lesion, **CL**: Sciatic nerve conditioning lesion, **GFP**: AAV5-GFP injection into L4 and L5 DRG. 0mm indicates caudal lesion edge. Animals where the retracting ‘leading edge’ was not visible within the sectioned 9mm caudal to the lesion were scored as 10mm (all but three animals). No significant differences were found between groups (Kruskal-Wallis test; n=5, 5 and 6).


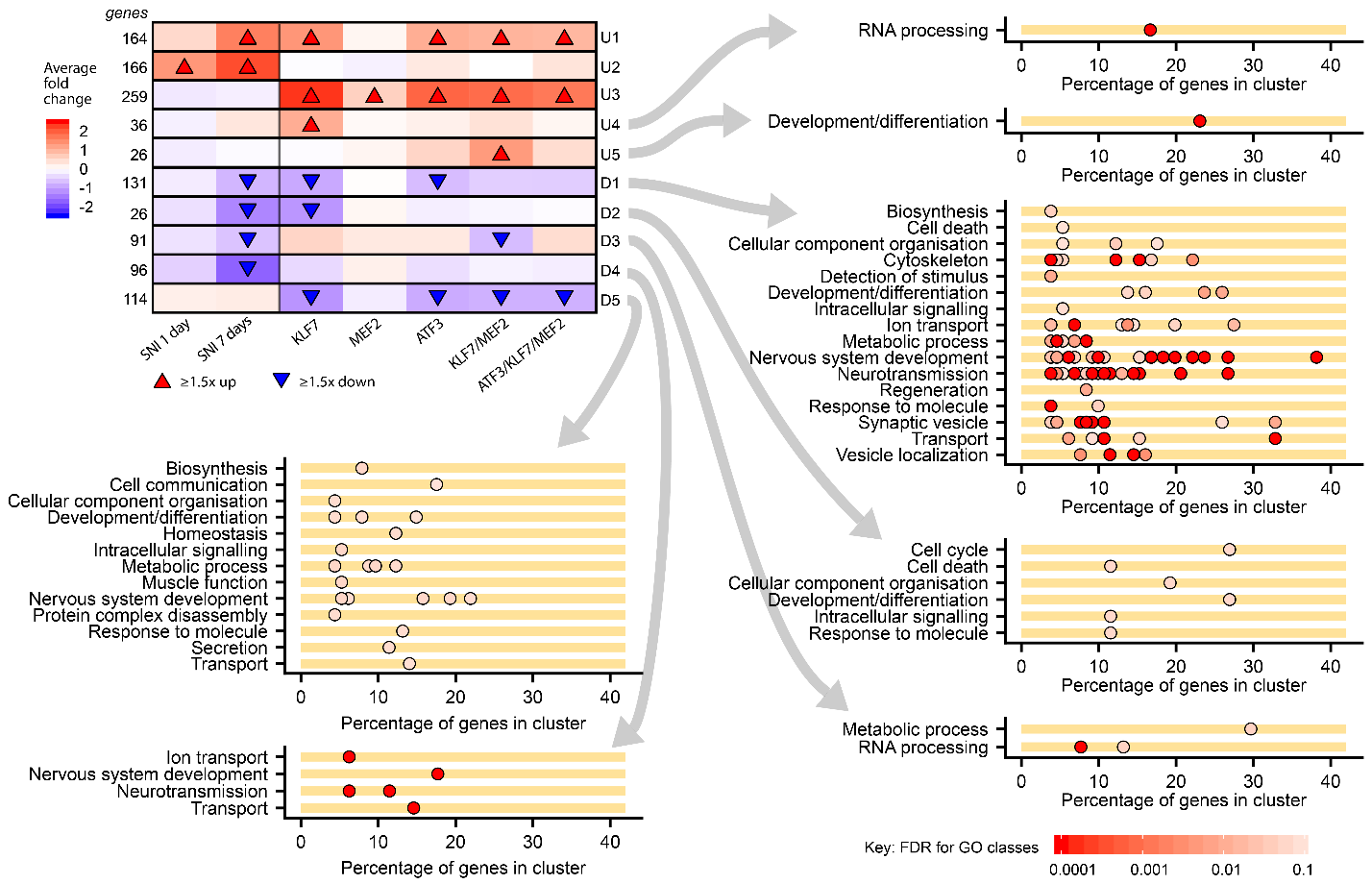


**Fig. S7.** Gene Ontology (GO) over-presentation analysis of the remaining clusters generated by Weighted Gene Correlation Network Analysis, not shown in Fig. 8. Cluster U4, containing genes induced only by KLF7 contains genes related to RNA processing. Cluster U5 is a small cluster of genes induced by KLF7/MEF2 and contains some genes related to development and differentiation. Clusters D1-D3 contain genes downregulated by axotomy and also by transcription factor groups. Of note D1 contains a number of genes related to neurotransmission and nervous system development, suggesting expression of KLF7 or ATF3 recapitulates this part of the axotomy-induced down-regulation of gene expression. Cluster D4 contains genes down-regulated by axotomy and not in any TF group, consisting of more genes related to neurotransmission and ion transport. Cluster D5 contains genes down-regulated by the TF groups (excluding MEF2) and not by axotomy, and includes genes related to nervous system development, cell communication and signalling.
