# Supplementary material for: The transcription factor combination MEF2 and KLF7 promotes axonal sprouting in the injured spinal cord with functional improvement and regeneration-associated gene expression
Source: Mol Neurodegener. 2025 Feb 8;20:18. doi: 10.1186/s13024-025-00805-4 (PMC11807332; doi:10.1186/s13024-025-00805-4)
Supplement: Supplementary file 4 — Supplementary Material 4 [file 13024_2025_805_MOESM4_ESM.docx]

| group | matrices | ratio | p | FDR | expressed | upregulated | other activation | factor chosen |
| --- | --- | --- | --- | --- | --- | --- | --- | --- |
| 1 | V$HAND1E47_01 | 5.1 | 0.00088 | 0.066 | Smad1, Smad2, Smad4, Smad5, Smad7, Tcf3 | Smad1 |  | Smad1EVE |
|  | PB0060: Smad3_1 | 3.8 | 0.00083 | 0.066 |  |  |  |  |
| 2 | V$T3R_01 | 4.5 | 4.7e-05 | 0.066 | Thra, Thrb |  |  |  |
| 3 | MA0119.1: TLX1::NFIC | 3.8 | 0.00016 | 0.066 | Nfic |  |  |  |
| 4 | V$GKLF_01 | 3.7 | 0.00058 | 0.066 | Klf2, Klf4, Klf6, Klf7 | Klf6 |  | Klf7 |
|  | MA0039.1: Klf4 | 3.4 | 4.4e-05 | 0.066 |  |  |  |  |
| 5 | V$STAT5B_01 | 3.7 | 0.00058 | 0.066 | Stat1, Stat3, Stat5a, Stat5b |  | Yes (Stat3) | Stat3C |
|  | MA0144.1: Stat3 | 3.7 | 0.00058 | 0.066 |  |  |  |  |
| 6 | MA0107.1: RELA | 3.7 | 0.0006 | 0.066 | Rela |  |  |  |
|  | V$NFKAPPAB65_01 | 3.5 | 0.00085 | 0.066 |  |  |  |  |
| 7 | V$MEF2_01 | 3.6 | 0.00042 | 0.066 | Mef2a |  | Yes | Mef2C-VP16 |
|  | V$MEF2_04 | 2.7 | 0.00093 | 0.068 |  |  |  |  |
| 8 | V$TBP_01 | 3.5 | 0.00081 | 0.066 | Tbp |  |  |  |
|  | PB0080: Tbp_1 | 3.5 | 6.1e-05 | 0.066 |  |  |  |  |
| 9 | PB0078: Srf_1 | 3.5 | 0.00081 | 0.066 | Srf |  | Yes | SRF-VP16 |
|  | V$SRF_01 | 3.1 | 0.0002 | 0.066 |  |  |  |  |
| 10 | V$TCF11MAFG_01 | 3.1 | 0.00041 | 0.066 | Mafg |  |  |  |
| 11 | V$AP1FJ_Q2 | 3.1 | 0.00021 | 0.066 | Atf3, Batf, Batf2, Batf3, Fos, Jdp2, Jun, Junb, Jund | Atf3, Jun |  | Atf3, Jun |
|  | V$AP1_Q4 | 3 | 3.8e-05 | 0.066 |  |  |  |  |
| 12 | V$RREB1_01 | 3 | 0.00076 | 0.066 | Rreb1 |  |  |  |
| 13 | V$CEBP_Q2 | 2.8 | 0.00065 | 0.066 | Cebpb, Cebpd, Cebpg, Ddit3 | Cebpd |  | Cebpd |
|  | MA0019.1: Ddit3::Cebpa | 2.6 | 0.00096 | 0.069 |  |  |  |  |
| 14 | V$E4BP4_01 | 2.7 | 9.5e-05 | 0.068 | Nfil3 | Nfil3 |  |  |
|  | MA0025.1: NFIL3 | 2.6 | 0.00021 | 0.069 |  |  |  |  |
| 15 | PB0165: Sox11_2 | 2.5 | 0.00077 | 0.069 | Sox11 | Sox11 |  | Sox11 |
| 16 | MA0109.1: Hltf | 2.5 | 0.00064 | 0.069 | Hltf |  |  |  |
| 17 | V$FOXO1_02 | 2.4 | 0.00021 | 0.069 | Foxj1, Foxo1 |  |  |  |
|  | PB0016: Foxj1_1 | 2.4 | 0.0001 | 0.069 |  |  |  |  |
| 18 | V$NFAT_Q6 | 2.4 | 9.4e-07 | 0.069 | Nfatc4 |  |  |  |
| 19 | PB0124: Gabpa_2 | 2.3 | 0.00096 | 0.071 | Gabpa |  |  |  |
| 20 | PB0033: Irf3_1 | 2.2 | 0.00083 | 0.073 | Irf3, Stat1 |  |  |  |
|  | MA0137.1: STAT1 | 2 | 0.0008 | 0.078 |  |  |  |  |
| 21 | PB0004: Atf1_1 | 2.2 | 1.9e-05 | 0.074 | Atf1, Atf3, Atf4, Atf5, Atf6, Atf6b, Creb1, Creb3, Crebbp, Crebl2, Crebzf, Crem | Atf3, Crem |  | (Atf3) |
| 22 | PB0028: Hbp1_1 | 2.1 | 1.1e-05 | 0.074 | Hbp1 |  |  |  |

**Supp. Table S1. Overrepresented transcription factor binding sites in the promoters of RAGs from mouse facial motor neurons (FMN) 1 day after axotomy.** TFBS motifs were used from TRANSFAC and JASPAR. The column ‘ratio’ gives the maximised ratio of frequency per kilobase of motif occurrence in RAG promoters compared to unregulated gene promoters. Each TFBS motif shown was at least 2-fold over-represented in RAG promoters compared to unregulated genes, with p<0.001 (binomial test). Motifs were considered only if at least one potentially binding TF was actually expressed in both mouse FMN and rat dorsal root ganglion (DRG) neurons (the factors meeting this criteria are listed in column ‘expressed’). Motifs were grouped together if they identified sites at the same locations (the top 2 motifs for each group are shown). TFs were further filtered according to whether either: they are upregulated after axotomy in both mouse FMN and rat DRG neurons (column ‘upregulated'); or a post-translational activation mechanism is known to exist, that is activated after axotomy (column ‘other activation’).

The post-translational activation mechanisms identified were as follows. STAT3 is known to be phosphorylated after axotomy (Schwaiger et al., 2000; Lee et al., 2004). MEF2 is regulated by histone deacetylases (HDACs) and HDAC deactivation and nuclear export has been demonstrated after axotomy (Potthoff and Olson, 2007; Cho et al., 2013). SRF is activated by phosphorylation of its transcriptional co-factor Elk1 after axotomy (Lin et al., 2003).

Nfil3 otherwise met the criteria for inclusion but was excluded because it has previously been shown to be inhibitory to regeneration (MacGillavry et al., 2009).

| group | matrix | ratio | p | FDR | expressed | upregulated | other activation | factor chosen |
| --- | --- | --- | --- | --- | --- | --- | --- | --- |
| 1 | V$MEF2_03 | 34 | 0.00019 | 0.05 | Mef2a |  | Yes | Mef2C-VP16 |
|  | V$MEF2_04 | 34 | 0.00019 | 0.05 |  |  |  |  |
| 2 | V$SRF_01 | 34 | 0.00019 | 0.05 | Srf |  | Yes | Srf-VP16 |
|  | V$SRF_C | 34 | 8.8e-10 | 0.05 |  |  |  |  |
| 3 | MA0019.1: Ddit3::Cebpa | 21 | 0.00063 | 0.057 | Ddit3 |  |  |  |
| 4 | MA0119.1: TLX1::NFIC | 17 | 0.00017 | 0.067 | Nfic |  |  |  |
| 5 | V$RREB1_01 | 16 | 0.00019 | 0.067 | Rreb1 |  |  |  |
| 6 | V$GKLF_01 | 13 | 0.00041 | 0.069 | Klf2, Klf4, Klf6, Klf7, Stat6 | Klf6 |  | Klf7 |
|  | MA0039.1: Klf4 | 11 | 0.00068 | 0.069 |  |  |  |  |
| 7 | PB0132: Hbp1_2 | 12 | 0.00048 | 0.069 | Hbp1 |  |  |  |
| 8 | MA0050.1: IRF1 | 9.9 | 0.00024 | 0.082 | Irf1, Irf7 |  |  |  |
|  | V$IRF7_01 | 3.5 | 0.0005 | 0.11 |  |  |  |  |
| 9 | PB0114: Egr1_2 | 8.8 | 0.0004 | 0.084 | Egr1 |  |  |  |
| 10 | PB0098: Zfp410_1 | 8.2 | 0.00053 | 0.085 | Zfp410 |  |  |  |
| 11 | V$TATA_01 | 7.4 | 2.5e-05 | 0.09 | Tbp |  |  |  |
|  | MA0108.1: TBP | 7.4 | 2.5e-05 | 0.09 |  |  |  |  |
| 12 | PB0038: Jundm2_1 | 7.1 | 1e-04 | 0.09 | Atf1, Atf3, Atf4, Atf5, Atf6, Atf6b, Creb1, Creb3, Crebbp, Crebl2, Crebzf, Crem, Jdp2, Mafb | Atf3, Crem |  | Atf3 |
|  | PB0004: Atf1_1 | 5.3 | 0.00054 | 0.1 |  |  |  |  |
| 13 | PB0186: Tcf3_2 | 4.7 | 0.00045 | 0.11 | Tcf3 |  |  |  |
| 14 | MA0144.1: Stat3 | 4.3 | 0.00041 | 0.11 | Stat3 | Stat3 | Yes | Stat3C |
| 15 | V$NFKAPPAB65_01 | 4.2 | 0.00094 | 0.11 | Rela |  |  |  |
| 16 | PB0003: Ascl2_1 | 3.9 | 0.00074 | 0.11 | Ascl2 |  |  |  |
| 17 | MA0116.1: Zfp423 | 3.6 | 0.00077 | 0.11 | Zfp423 |  |  |  |
| 18 | V$MAX_01 | 3.4 | 0.00034 | 0.11 | Max |  |  |  |
|  | MA0058.1: MAX | 3.3 | 0.00051 | 0.11 |  |  |  |  |
| 19 | PB0025: Glis2_1 | 3.2 | 2.7e-05 | 0.11 | Glis2 |  |  |  |
| 20 | PB0156: Plagl1_2 | 3.2 | 0.00024 | 0.11 | Plagl1 |  |  |  |
| 21 | PB0196: Zbtb7b_2 | 3.1 | 0.00084 | 0.11 | Zbtb7b |  |  |  |
| 22 | MA0105.1: NFKB1 | 3 | 0.00058 | 0.11 | Nfkb1 |  |  |  |
| 23 | PB0009: E2F3_1 | 2.4 | 0.00061 | 0.11 | E2f3 |  |  |  |

**Supp. Table S2. Overrepresented transcription factor binding sites in the promoters of upregulated transcription factors in mouse facial motor neurons (FMN) 1 day after axotomy.** The expressed, upregulated and other activation columns are as in Supp. Table S1. In this analysis only the promoters of transcription factors were considered, so this analysis is designed to identify TFs that regulate other TFs after axotomy and thus are likely to be hub TFs. Of note the over-representation ratios are much higher in this analysis, suggesting it has higher sensitivity than considering all RAGs. Furthermore MEF2 and SRF score particularly strongly suggesting one or both may be key hub genes in the RAG program.

| **Experiment** | **Outcome measures** | **Groups** | **Initial n** | **Exclusions/** | **Final n** |
| --- | --- | --- | --- | --- | --- |
|  |  |  |  | **losses** |  |
| AAV-TF/GFP delivery to DRG with spinal cord injury | Axon sprouting by immunohistochemistry  Functional testing (horizontal ladder, inclined rolling ladder, CatWalk) | No-TF/GFP/sham lesion | 9 |  | 9 |
|  |  | No-TF/GFP |  | 1 euthanased early | 8 |
|  |  | MEF2/GFP |  | 1 euthanased early | 8 |
|  |  | KLF7/GFP |  |  | 9 |
|  |  | KLF7/MEF2/GFP |  |  | 9 |
|  |  | ATF3/ KLF7/MEF2/GFP |  |  | 9 |
| AAV-TF/fluorophore delviery to DRG for immunohistochemistry | Immunohistochemistry | ATF3/GFP | 3 |  | 3 |
|  |  | KLF7/GFP |  |  | 3 |
|  |  | MEF2/GFP |  |  | 3 |
| AAV-TF/fluorophore delivery to DRG for gene expression profiling | Gene expression profiles | No-TF/GFP | 4 |  | 4 |
|  |  | ATF3/mitoYFP |  |  | 4 |
|  |  | MEF2/GFP |  | 1 excluded | 3 |
|  |  | KLF7/mCherry |  |  | 4 |
|  |  | MEF2/GFP + KLF7/mCherry |  |  | 4 |
|  |  | MEF2/GFP + KLF7/mCherry + ATF3/mitoYFP |  |  | 4 |
| Sciatic nerve injury for gene expression profiling | Gene expression profiles | Naïve | 4 |  | 4 |
|  |  | Sciatic nerve injury (1 day survival) |  |  | 4 |
|  |  | Sciatic nerve injury (7 day survival) |  |  | 4 |
| Dorsal column lesion with conditioning lesion and/or AAV-TF/GFP delivery to DRG | Axon tracing/retraction measurement | AAV-GFP + conditioning lesion + dorsal column lesion | 6 |  | 6 |
|  |  | conditioning lesion + dorsal column lesion |  |  | 6 |
|  |  | dorsal column lesion only |  |  | 6 |

**Supp. Table S3. Summary of experimental animal groups used.** Numbers in each group are given and where animals were euthanased early or excluded this is indicated.

| Motif | Genes |
| --- | --- |
| AP1 | 1810062O18Rik, Nid67, RGD1560812, Npepo, 2210403K04Rik, 4930512H18Rik, ENSRNOG00000023765, ENSRNOG00000018126, Abhd2, Acsl4, Adam9, Adamts5, Adcyap1, Agfg1, Ankrd1, Arpc5, Arrdc4, Atf1, Atf3, Calca, Camk4, LOC685173, Ccdc80, Ccl2, Ccl7, LOC685808, Cd24, Cd3eap, Cd44, Clp1, Col7a1, LOC689288, Csf1, Dusp6, Eaf1, Eif4e, Emp1, Emp3, Erc2, Etv5, Fam108b1, Fgf3, Peg12, Fst, Gadd45a, Gap43, Gfra1, Gpr158, Gpr183, Gpr19, ENSRNOG00000021058, Hbegf, Hk2, Hs2st1, Htr2b, Ier5, Il1r1, Il6, Inhba, Ipo5, Itpr1, Jun, Kera, Klf7, Lamc2, Lix1, Lrfn4, Lrrfip2, Lrtm2, Ly96, Mafk, ENSRNOG00000007053, Midn, Morc2, Ndst1, Nov, Nppb, Nras, P2ry6, Plagl1, Plaur, Plekho1, Plk3, Procr, LOC100365697, Ptpn1, Rap1b, Rchy1, Rdh10, Rhoj, Rhoq, Rras2, S100a16, Sat1, Sec16b, Serpinb2, Sertad1, Slc39a14, Slitrk6, Socs3, Spp1, ENSRNOG00000031167, Stam, Stat5b, Syt12, ENSRNOG00000011238, Tm4sf1, Tpm4, Ttc9, Txk, Txn1, Uap1, ENSRNOG00000025697, Uck2, Vash2, Vim, Vps4b, Wbp5, Wsb1, LOC691024, RGD1309228, 4930512H18Rik, ENSRNOG00000038119, ENSRNOG00000018126, Acads, Adamts5, LOC366473, Agfg1, Ankrd1, Ankrd23, LOC305633, Anxa10, Apobec2, Arid3a, Arpc5, Arrdc4, Brms1, ENSRNOG00000011331, Cacna2d1, Calca, LOC685173, Ccdc80, Ccl7, LOC685808, Cd34, Cd44, Cd68, Cd96, Cdkn2b, Cndp2, Coro2a, Crct1, Cryba2, Csf1r, RGD1306959, Dhrs2, Dusp6, Eef1b2, Eif4e2, Elovl1, Erc2, Erlin1, Ext2, F11r, Fgf3, Fli1, Frmd3, Fst, Gap43, ENSRNOG00000040025, Kiss1, Gpr158, Grem2, ENSRNOG00000039629, Grrp1, ENSRNOG00000021058, Hcls1, Hk2, Hpgd, Hsd17b7, Hspb3, Il1r1, Il6, Inpp5d, Jun, Kera, Kif3b, Klf7, Ka11, Lix1, Lpxn, Lrrc25, Lrtm2, Ly96, LOC100359928, Mapk3, Mapk6, Met, Myh1, Myom2, Nckap1l, Ndst1, Nov, Npy, Nrip1, Nrp2, Obfc1, P2ry6, Phex, Plaur, Plscr3, Pmaip1, Ppp2r5b, ENSRNOG00000002004, Prkcdbp, Procr, LOC100365697, Ptprc, Pvrl1, Rap1b, Rdh10, Rela, Rhoq, Rin1, Riok3, Rnd1, Rtn4, RGD1563581, ENSRNOG00000021015, Sdcbp2, Selplg, Serpinb2, Serpinb5, Sertad1, Sfrp1, Siglec5, Sipa1l2, Slc35e4, Slc6a4, Slitrk6, Sncb, Sprr1a, Srpx2, ENSRNOG00000031167, Star, Stmn4, Tbxas1, Tm4sf1, Trem2, Uap1, Vash2, Vim, Vip, Wars, Wbp5, Zfp367 |
| KLF | 1810062O18Rik, RGD1560812, 2210403K04Rik, ENSRNOG00000023765, Adcyap1, Angptl2, Atf3, Azin1, Bend5, Ccdc80, Cited2, Clec5a, ENSRNOG00000037794, Nrep, Dusp6, Elk3, Fads3, Gadd45g, Gpr19, LOC100364712, Heca, Hgf, Hk2, Inhbb, Jun, Klf7, Lpo, Lrrc3b, ENSRNOG00000023527, Myc, Ndst1, ENSRNOG00000033527, Pcsk1, Pdgfc, Pik3r6, Procr, S100a16, Sertad1, Snai2, Socs3, Ttc9, Ubash3b, Wbp5, Znf710 |
| MEF2 | 1810011O10Rik, 1810062O18Rik, Acvr1, Adcyap1, Ankrd1, Arpc5, Arrdc4, Atf3, Azin1, Bend5, Btg2, Camkk2, Ccdc80, Chmp1b, Cited4, Clp1, Csf2rb, Ctsc, Nrep, Ebp, Egr1, Ephb1, LOC498276, Flrt3, Gmds, Gpr19, Grp, Grrp1, LOC100364712, Hbegf, Hk2, Hnrnpab, Htr2b, Idh1, Ier5, Inhba, Itpr1, Jun, Klf6, Klf7, March6, Med8, Mlf1, Morc2, Myadm, Myc, Myo18b, Nov, Nras, ENSRNOG00000033527, Pcnx, Plcd1, Pnlip, Prrc1, Rchy1, Rg9mtd2, Riok3, Rras2, Sema3e, Slamf9, Slc7a8, Slitrk6, Smad1, Snai2, Sox11, Spata13, Spata5, Spp1, Stat5b, Steap1, Syt4, ENSRNOG00000037329, Timm8a1, Ttc9, Ubash3b, Vim, Vip, Wbp5, Wdr1, Wsb1, ENSRNOG00000022686, 1110038B12Rik, LOC361776, RGD1560812, 5033430I15Rik, ENSRNOG00000018126, ENSRNOG00000003538, Anxa10, Apobec2, Atf3, Azin1, Bend5, RGD1564541, Ccdc80, Cd180, ENSRNOG00000019851, Crym, Csf2rb, Ctsc, Cx3cr1, Ebp, Ephb1, Fli1, Flrt3, Gal, Galnt3, Gp9, LOC689257, Gpr34, Hist1h1c, Hist1h1d, Hist1h2aa, LOC100360053, Hist1h2bm, Hk2, Hsd17b7, Hspb3, Igsf6, Itpr1, Jun, Klf6, Klf7, Klhl6, Kpna3, Krtap17-1, Lipi, Lrch4, Lyl1, Mapk14, Myh1, Myo10, Myo18b, Myom2, P2ry12, Phex, Plcd1, Pnlip, Prkg2, Ptprc, Rela, Rhov, Rras2, Sfrp1, Slamf9, Slc6a4, Sln, Slpi, Smad1, Sox11, Sprr1a, Sri, Steap1, Tbxas1, Tnfaip8l2, Tnfrsf13b, Vat1, Vip, Wdr1, Zbtb7c |

**Supp. Table S4. Genes upregulated after axotomy whose promoters contain transcription factor binding site motifs for AP1, KLF family and MEF2 family TFs.** These genes are thus putative targets of the TFs ATF3 (when dimerized with c-Jun), KLF7 and MEF2.

**References**

Cho Y, Sloutsky R, Naegle KM, Cavalli V (2013) Injury-Induced HDAC5 nuclear export is essential for axon regeneration. Cell 155:894–908 Available at: http://dx.doi.org/10.1016/j.cell.2013.10.004.

Lee N, Neitzel KL, Devlin BK (2004) STAT3 Phosphorylation in Injured Axons before Sensory and Motor Neuron Nuclei : Potential Role for STAT3 as a Retrograde Signaling Transcription Factor. 545:535–545.

Lin H, Bao J, Ying J, Walters ET, Ambron RT (2003) Rapid electrical and delayed molecular signals regulate the serum response element after nerve injury: Convergence of injury and learning signals. J Neurobiol 57:204–220 Available at: http://doi.wiley.com/10.1002/neu.10275.

MacGillavry HD, Stam FJ, Sassen MM, Kegel L, Hendriks WTJ, Verhaagen J, Smit AB, van Kesteren RE (2009) NFIL3 and cAMP Response Element-Binding Protein Form a Transcriptional Feedforward Loop that Controls Neuronal Regeneration-Associated Gene Expression. J Neurosci 29:15542–15550.

Potthoff MJ, Olson EN (2007) MEF2: a central regulator of diverse developmental programs. Development 134:4131–4140 Available at: http://dev.biologists.org/cgi/doi/10.1242/dev.008367.

Schwaiger F, Hager G, Schmitt AB, Horvat A, Hager G, Streif R, Spitzer C, Gamal S, Breuer S, Brook GA, Nacimiento W, Kreutzberg GW (2000) Peripheral but not central axotomy induces changes in Janus kinases ( JAK ) and signal transducers and activators of transcription ( STAT ). 12:1165–1176.
